# Supplementary material for: LKB1 drives stasis and C/EBP-mediated reprogramming to an alveolar type II fate in lung cancer
Source: Nat Commun. 2022 Feb 28;13:1090. doi: 10.1038/s41467-022-28619-8 (PMC8885825; doi:10.1038/s41467-022-28619-8)
Supplement: Supplementary file 1 — Supplementary Information [file 41467_2022_28619_MOESM1_ESM.pdf]

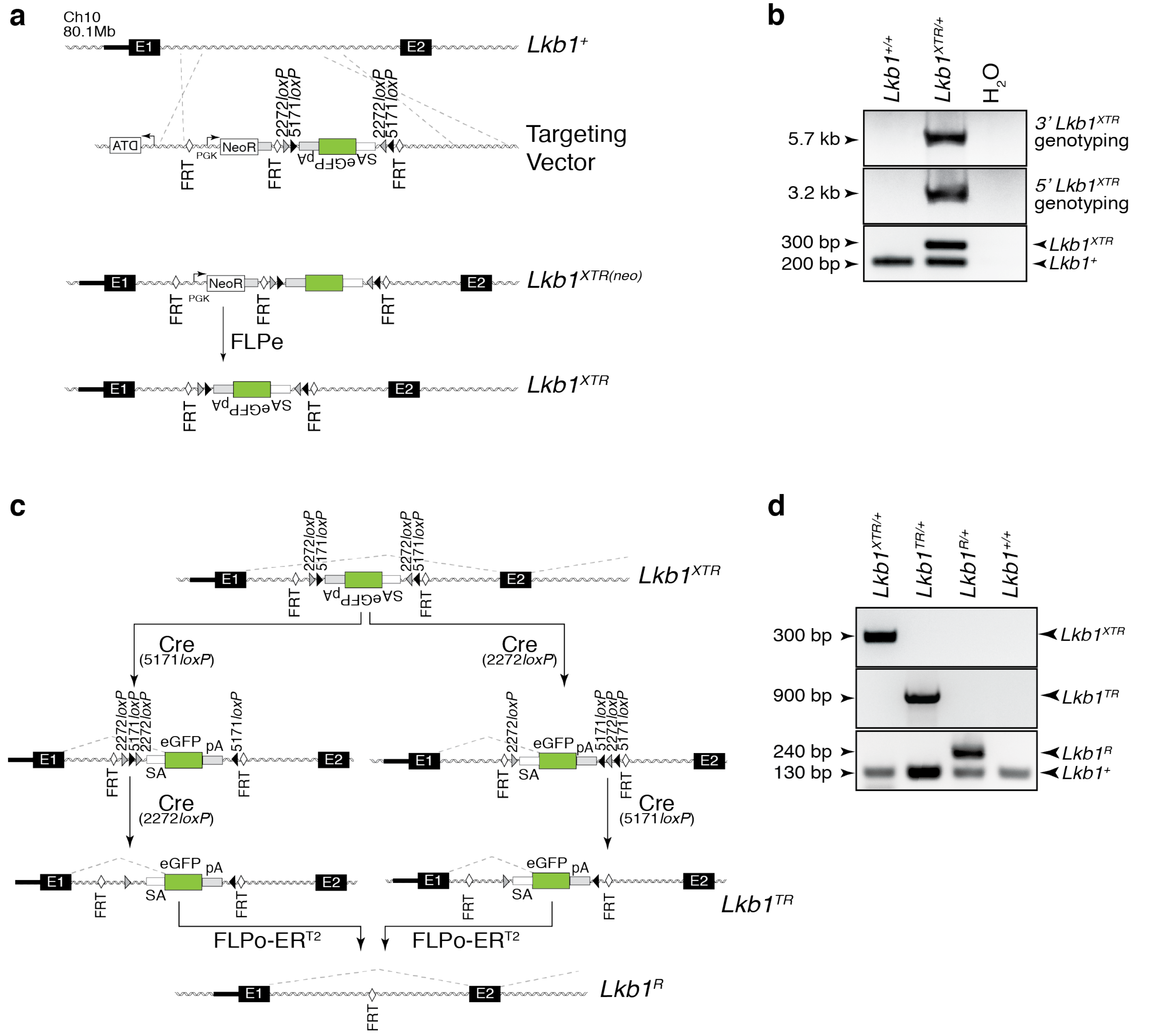

Supplementary Figure 1. Generation of a conditionally inactivatable and restorable *Lkb1*^{XTR} allele.

a. Design of targeting vector to insert the XTR cassette into the first intron of *Lkb1*. Proper homologous recombination in ES cells generated the *Lkb1*$^{XTR(neo)}$ allele, which encodes neomycin resistance (NeoR) to enable selection of ES cells harboring integrations of the targeting vector. *Lkb1*$^{XTR(neo)/+}$ ES cells were used to generate chimeric mice to enable germline transmission. *Lkb1*$^{XTR(neo)/+}$ mice were crossed with *R26*^{FLPe} mice. In *Lkb1*$^{XTR(neo)/+}$;*R26*$^{FLPe/+}$ mice, FLPe is expressed at sufficiently low levels to enable partial recombination of the three FRT sites in the *Lkb1*$^{XTR(neo)}$ allele, thus generating a subset of offspring with the intact *Lkb1*^{XTR} allele. SA = Ad40 splice acceptor. eGFP = enhanced GFP. pA = SV40 polyA. NeoR = neomycin resistance, positive selection marker. DTA = diptheria toxin A, negative selection marker.

b. Long-range PCR analysis (top) and standard genotyping (bottom) of mice with the *Lkb1*^{XTR} allele.

c. Two sequential Cre-mediated recombination events between pairs of head-to-head oriented, heterotypic *loxP* sites leads to stable inversion of the XTR cassette, thus converting the *Lkb1*^{XTR} allele from its expressed conformation to its trapped *Lkb1*^{TR} conformation. These two recombination events can occur with Cre acting on the *5171loxP* sites first and then *2272loxP* sites second (left arrows) or vice versa (right arrows). Subsequent FLP-mediated recombination of the FRT sites deletes the SA-GFP cassette, thus converting the *Lkb1*^{TR} allele to the restored *Lkb1*^{R} conformation. Anticipated splicing from exon 1 to exon 2 in the *Lkb1*^{XTR} and *Lkb1*^{R} alleles, and from Exon 1 to GFP are indicated by dashed lines.

d. PCR genotyping of *Lkb1*$^{XTR/+}$ MEFs upon conversion to the *Lkb1*^{TR} and subsequent *Lkb1*^{R} states mediated by Adeno-Cre and Adeno-FLP, respectively. *Lkb1*$^{+/+}$ MEFs are shown as a negative control.

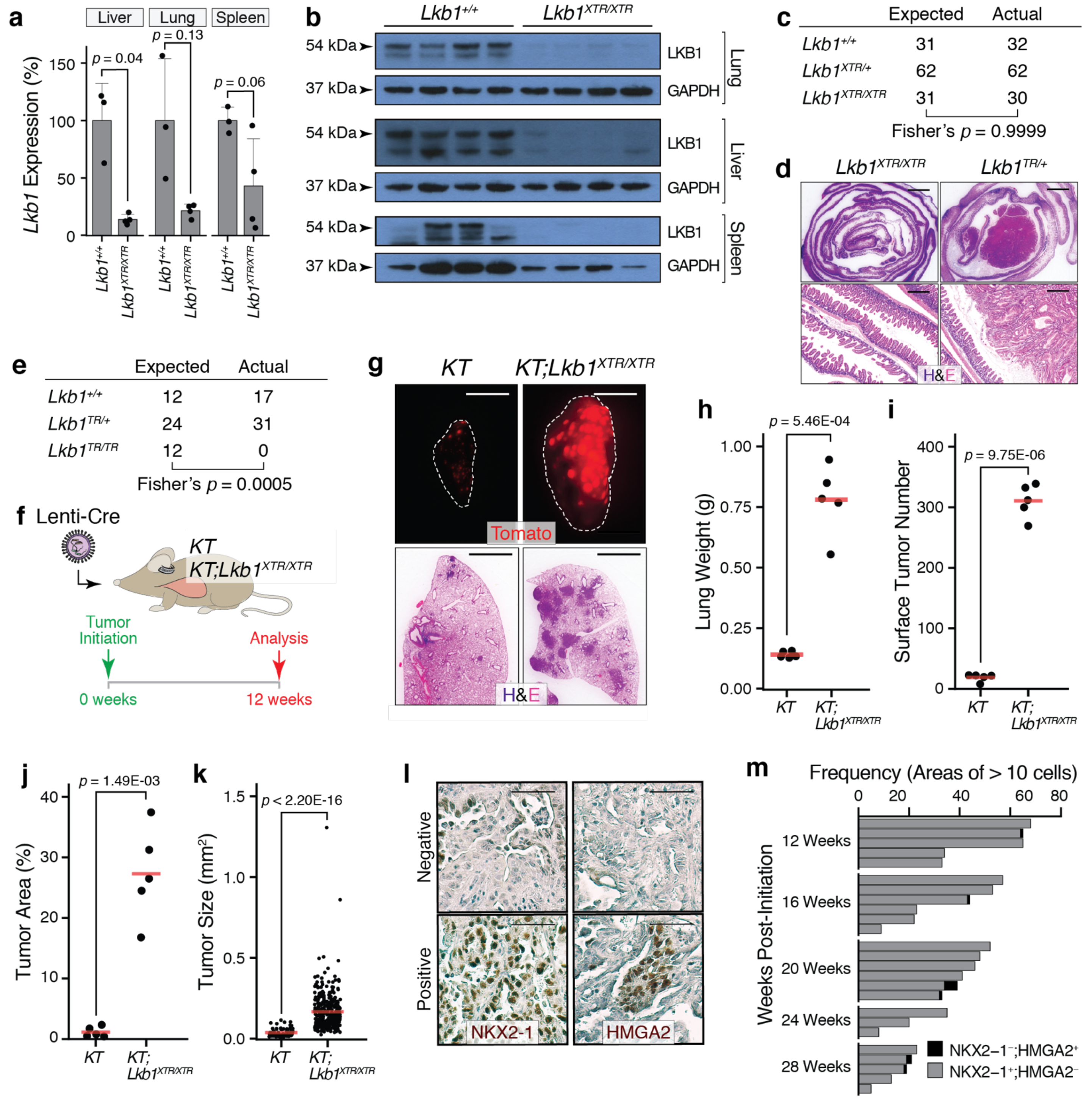

Supplementary Figure 2. Functional characterization of the *Lkb1*^{XTR} allele in the expressed and trapped conformations.

a. *Lkb1* mRNA abundance measured by qRT-PCR in *Lkb1*$^{+/+}$ ($n = 3$ mice) and *Lkb1*$^{XTR/XTR}$ ($n = 4$ mice) mice. Bars and error bars indicate the mean and standard deviation, respectively. Normalized to *Lkb1*$^{+/+}$ samples and *Gapdh* housekeeping control. *P* values were calculated by two-sided unpaired t-test.

b. Western blot for LKB1 protein levels in *Lkb1*$^{+/+}$ and *Lkb1*$^{XTR/XTR}$ mice. GAPDH shows loading. $N = 4$ mice per genotype. Images were acquired from a single experiment including multiple biological replicates.

c, e. Frequency of genotypes yielded from *Lkb1*$^{XTR/+}$ (**c**) and *Lkb1*$^{TR/+}$ (**e**) intercrosses.

d. Small intestines from adult *Lkb1*$^{TR/+}$ (average age of 38 weeks) and *Lkb1*$^{XTR/XTR}$ (average age of 51 weeks) mice. Intestinal polyps were present in aged *Lkb1*$^{TR/+}$ mice and not in *Lkb1*$^{XTR/XTR}$ mice (0/10 mice). Bottom, higher magnification images. Top scale bars = 5 mm. Bottom scale bars = 500 µm. Images were acquired from a single experiment including multiple biological replicates.

f. Lung tumor initiation in *KT* and *KT;Lkb1*$^{XTR/XTR}$ mice mediated by intratracheal delivery of lentiviral Cre. Tumor burden was assessed after 12 weeks.

g. Fluorescence (top) and hematoxylin-eosin (H&E) staining (bottom) images of tumor-bearing lungs. Lung lobes are outlined in white. Top scale bars = 5 mm. Bottom scale bars = 2 mm.
h. Total lung weight for tumor-bearing mice. Each dot represents a mouse and red crossbars indicate the mean. $N = 5$ mice per genotype. P values were calculated by two-sided unpaired t-test.
i-k. Number of tdTomato^{positive} surface tumors (>1 mm in diameter by fluorescence, **i**), tumor area (**j**) and size (**k**) from histological sections of tumor-bearing lungs. Each dot represents either a mouse (**i** and **j**) or a tumor (**k**). Red crossbars indicate the mean. $N = 5$ mice per genotype. One tissue section per mouse was analyzed. In **k**, *KT*, $n = 77$ tumors; *KT;Lkb1*$^{XTR/XTR}$, $n = 318$ tumors. P values were calculated by two-sided unpaired t-test.
l. Immunohistochemical staining for NKX2-1 and HMGA2 within tumor-bearing lungs of *KT;Lkb1*$^{XTR/XTR}$ mice. Images were acquired from a single experiment including multiple biological replicates as noted in **m**.
m. Incidence of NKX2-1^{+};HMGA2^{-} and NKX2-1^{-};HMGA2^{+} areas within lung tumors of *KT;Lkb1*$^{XTR/XTR}$ mice across several time points following tumor initiation. One tissue section per mouse was analyzed. 12 weeks, $n = 5$ mice; 16 weeks, $n = 6$ mice; 20 weeks, $n = 6$ mice; 24 weeks, $n = 3$ mice; 28 weeks, $n = 5$ mice.

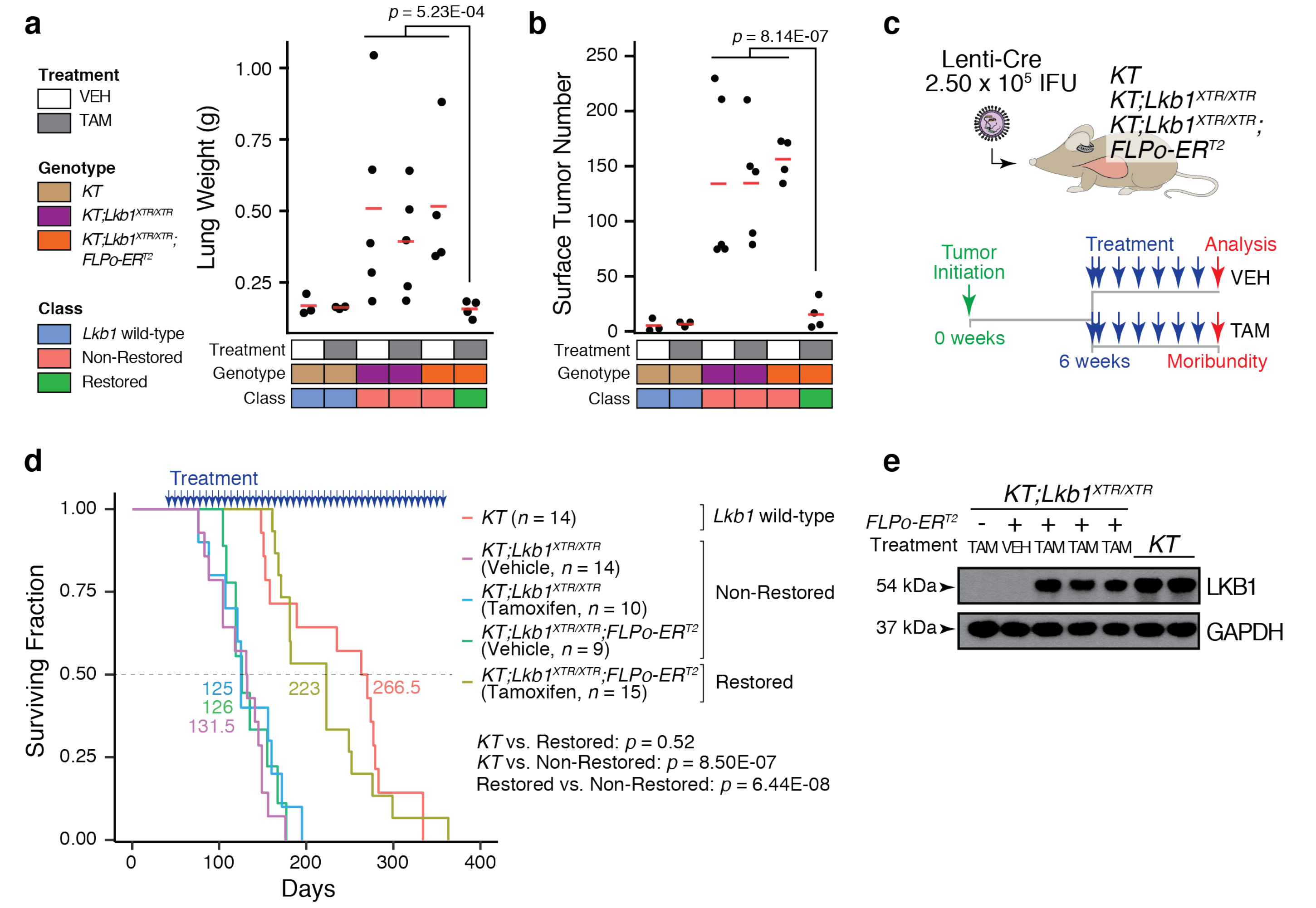

Supplementary Figure 3. *Lkb1* restoration decreases lung tumor burden and extends survival.

a, b. Total lung weight (**a**) and tdTomato^{positive} surface tumor number (**b**) for tumor-bearing *KT*, *KT;Lkb1*$^{XTR/XTR}$, and *KT;Lkb1*$^{XTR/XTR}$*;FLPo-ER*^{T2} mice at 12 weeks after tumor initiation, following six weeks of treatment with vehicle or tamoxifen. Each dot represents an individual mouse. *KT*-vehicle, *n* = 3 mice; *KT*-tamoxifen, *n* = 3 mice; *KT;Lkb1*$^{XTR/XTR}$-vehicle, *n* = 5 mice; *KT;Lkb1*$^{XTR/XTR}$-tamoxifen, *n* = 5 mice; *KT;Lkb1*$^{XTR/XTR}$*;FLPo-ER*^{T2}-vehicle, *n* = 4 mice; *KT;Lkb1*$^{XTR/XTR}$*;FLPo-ER*^{T2}-tamoxifen, *n* = 4 mice. Red crossbars indicate the mean. *P* values were calculated by two-sided unpaired t-test. VEH vehicle. TAM tamoxifen.

c. Survival analysis to capture the impact of *Lkb1* restoration on the longevity of lung tumor-bearing mice. Tumors were initiated in *KT*, *KT;Lkb1*$^{XTR/XTR}$, and *KT;Lkb1*$^{XTR/XTR}$*;FLPo-ER*^{T2} mice via intratracheal delivery of Lenti-Cre. At six weeks post-initiation, tumor-bearing mice began weekly treatment with vehicle or tamoxifen. IFU infectious units.

d. Kaplan-Meier survival analysis of mice harboring *Lkb1* wild-type, non-restored, and restored tumors. *KT*, *n* = 14 mice; *KT;Lkb1*$^{XTR/XTR}$-vehicle, *n* = 14 mice; *KT;Lkb1*$^{XTR/XTR}$-tamoxifen, *n* = 10 mice; *KT;Lkb1*$^{XTR/XTR}$*;FLPo-ER*^{T2}-vehicle, *n* = 9 mice; *KT;Lkb1*$^{XTR/XTR}$*;FLPo-ER*^{T2}-tamoxifen, *n* = 15 mice. Median survival for each cohort is indicated in colored text. *P* values determined using log-rank test.

e. Western blot analysis of LKB1 protein abundance in neoplastic cells sorted from *Lkb1* wild-type, non-restored, and restored tumors. ACTIN shows loading. Images were acquired from a single experiment including multiple biological replicates. TAM tamoxifen. VEH corn oil vehicle. “+” FLPo-ER^{T2} -proficient. “-“ FLPo-ER^{T2} -deficient.

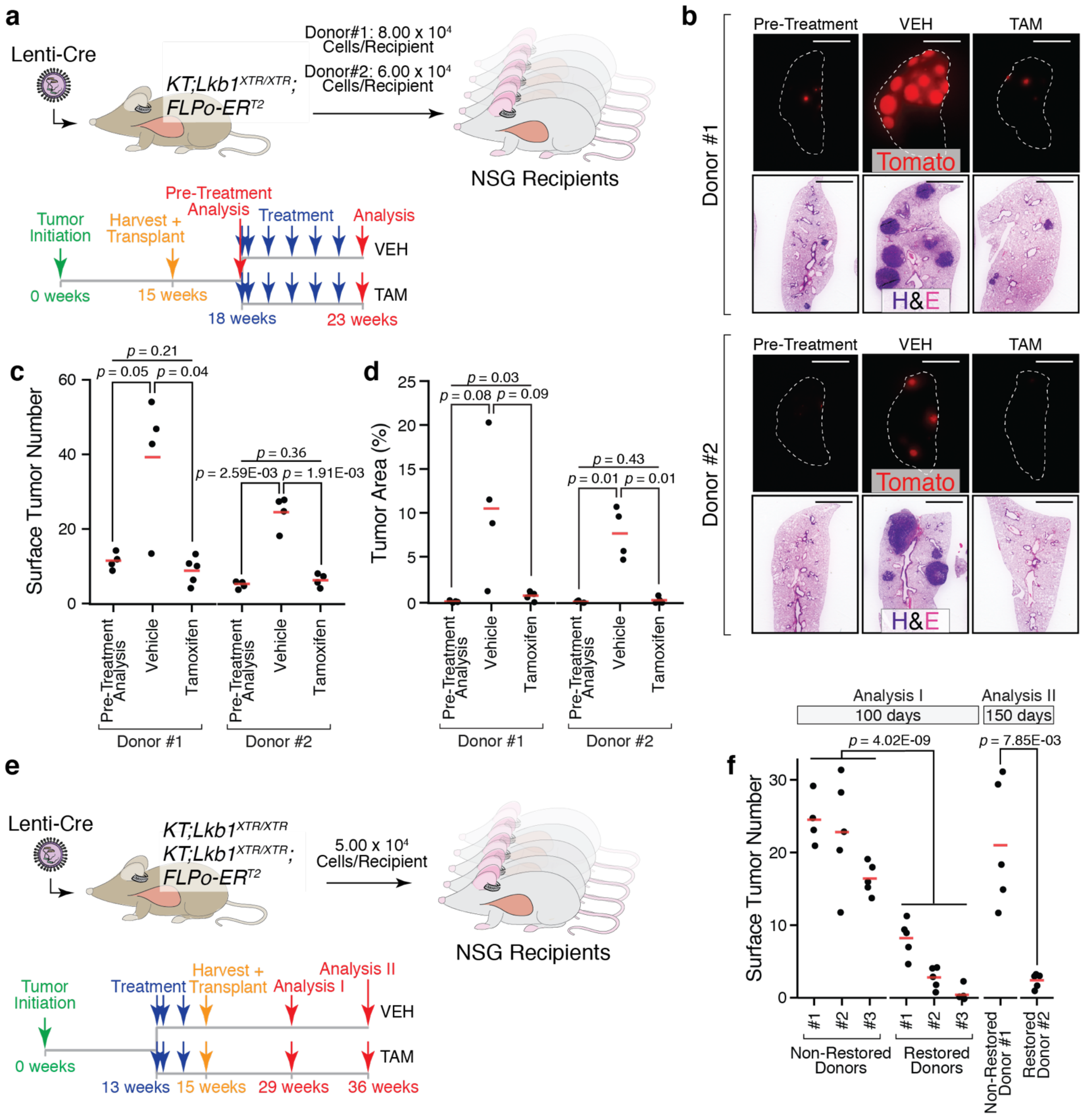

Supplementary Figure 4. Restoration of *Lkb1* impairs lung tumor formation and outgrowth.

a. The *Lkb1*^{XTR} allele in its expressed conformation is hypomorphic (**Supplementary Fig. 2a, b**), thus FLPo-ER^{T2} mediated excision of the gene trap within non-neoplastic cells presumably leads to a global increase in *Lkb1* expression. It is important to assess the neoplastic cell response to *Lkb1* restoration in the context of constant *Lkb1* expression within non-neoplastic compartments. Neoplastic cells were harvested from *KT;Lkb1*$^{XTR/XTR}$*;FLPo-ER*^{T2} donor mice at 15 weeks post-initiation and transplanted into the lungs of NOD/SCID/γc (NSG) mice via intratracheal delivery. At three weeks post-transplant, a subset of recipients were analyzed, while the remaining were treated with vehicle or tamoxifen for five weeks prior to analysis.

b. Representative fluorescence (top) and hematoxylin-eosin (H&E) staining (bottom) images of tumor-bearing lungs from NSG recipient mice at treatment initiation or following treatment. Lung lobes are outlined in white. Top scale bars = 5 mm. Bottom scale bars = 2 mm. Images were acquired from a single experiment including multiple biological replicates. VEH vehicle. TAM tamoxifen.

c, d. Number of tdTomato^{positive} surface tumors (**c**) detected by fluorescence microscopy and tumor area (**d**) as assessed by histology of tumor-bearing lungs from recipient mice. Each dot represents a mouse and red crossbars indicate the mean. Donor#1-Pre-Treatment, n = 4 recipients; Donor#1-vehicle, n = 4 recipients; Donor#1-tamoxifen, n = 5 recipients; Donor#2-Pre-Treatment, n = 4 recipients; Donor#2-

vehicle, *n* = 4 recipients; Donor#2-tamoxifen, *n* = 4 recipients. One tissue section per mouse was analyzed. *P* values were calculated by two-sided unpaired t-test.

e. Neoplastic cells were harvested from *KT;Lkb1*$^{XTR/XTR}$ and *KT;Lkb1*$^{XTR/XTR}$*;FLPo-ER*^{T2} donor mice at 15 weeks post-initiation, following two weeks of tamoxifen treatment, and transplanted into the lungs of NSG mice via intratracheal delivery. Lung tumor burden within recipients was assessed at 14 and 21 weeks post-transplant. VEH vehicle. TAM tamoxifen.

f. Number of tdTomato^{positive} surface lung tumors in recipient mice. Each dot represents a mouse and red crossbars indicate the mean. Non-Restored Donor#1, *n* = 4 recipients; Non-Restored Donor#2, *n* = 5 recipients; Non-Restored Donor#3, *n* = 5 recipients; Restored Donor#1, *n* = 5 recipients; Restored Donor#2, *n* = 5 recipients; Restored Donor#3, *n* = 5 recipients; Non-Restored Donor#1 (late), *n* = 5 recipients; Restored Donor#2 (late), *n* = 5 recipients. *P* values were calculated by two-sided unpaired t-test.

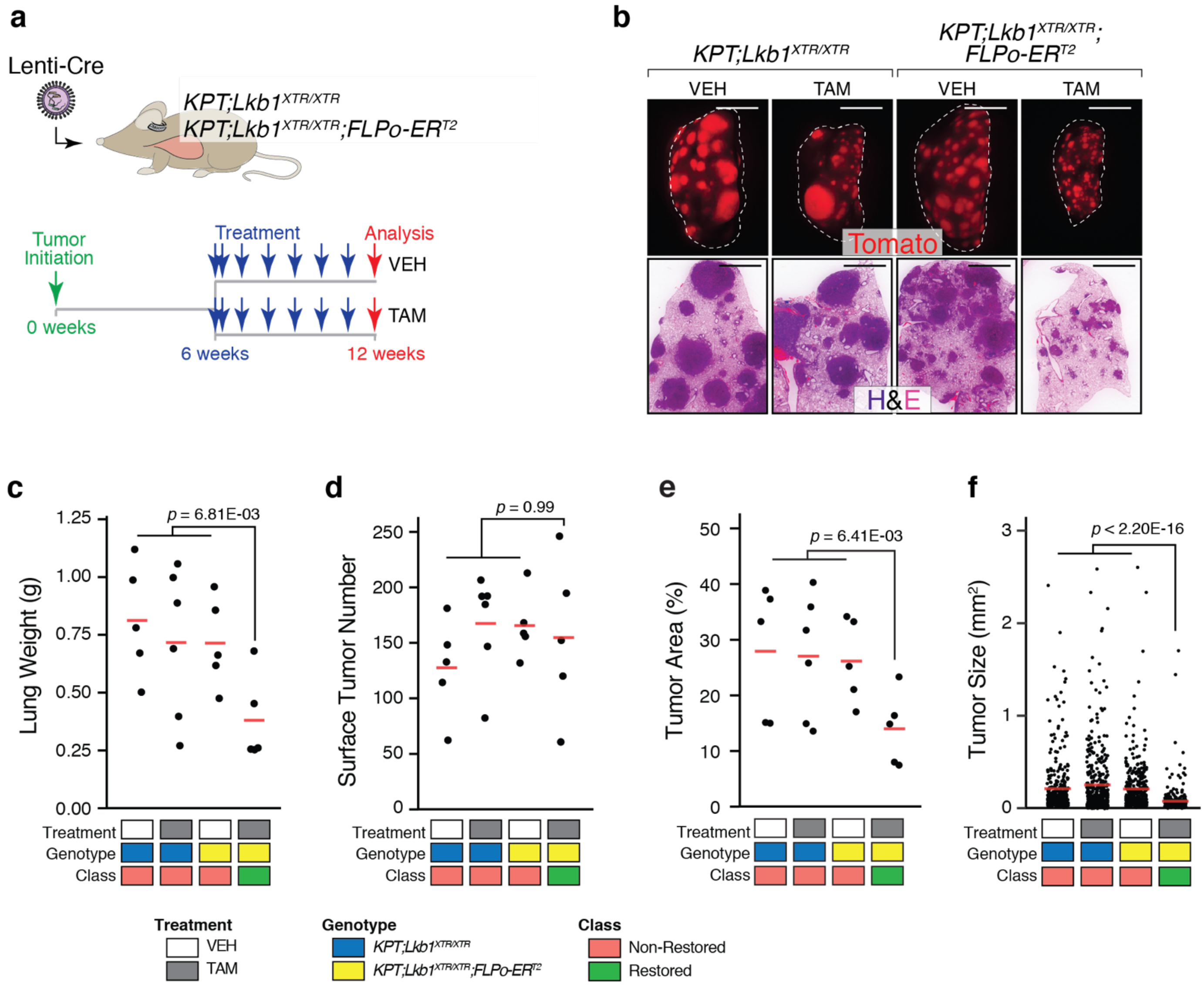

Supplementary Figure 5. *Lkb1* restoration decreases lung tumor burden in the absence of p53.

a. Tumor study to determine whether *Trp53* inactivation abrogates the growth-suppressive effect of *Lkb1* restoration. Tumors were initiated in *KT;Trp53$^{flox/flox}$* (KPT)*;Lkb1$^{XTR/XTR}$*, and *KPT;Lkb1$^{XTR/XTR}$;FLPo-ER^{T2}* mice via intratracheal delivery of lentiviral Cre. At six weeks post-initiation, tumor-bearing mice were treated weekly for six weeks with vehicle or tamoxifen prior to analysis of tumor burden. VEH vehicle. TAM tamoxifen.

b. Representative fluorescence (top) and hematoxylin-eosin (H&E) staining (bottom) images of tumor-bearing lungs from *KPT;Lkb1$^{XTR/XTR}$*, and *KPT;Lkb1$^{XTR/XTR}$;FLPo-ER^{T2}* mice treated with either vehicle or tamoxifen as indicated. Lung lobes within fluorescent images are outlined in white. Top scale bars = 5 mm. Bottom scale bars = 2 mm.

c-f. Total lung weight (**c**), number of tdTomato^{positive} surface tumors (>1 mm in diameter, **d**) detected by fluorescence microscopy, as well as tumor area (**e**) and size (**f**) as assessed by histology of tumor-bearing lungs from *KPT;Lkb1$^{XTR/XTR}$*, and *KPT;Lkb1$^{XTR/XTR}$;FLPo-ER^{T2}* mice treated with vehicle or tamoxifen. In **c-e**, each dot represents a mouse, whereas each dot corresponds to a tumor in **f**. *KPT;Lkb1$^{XTR/XTR}$*-vehicle, n = 5 mice; *KPT;Lkb1$^{XTR/XTR}$*-tamoxifen, n = 6 mice; *KPT;Lkb1$^{XTR/XTR}$;FLPo-ER^{T2}*-vehicle, n = 5 mice; *KPT;Lkb1$^{XTR/XTR}$;FLPo-ER^{T2}*-tamoxifen, n = 5 mice. In **f**, *KPT;Lkb1$^{XTR/XTR}$*-vehicle, n = 360 tumors; *KPT;Lkb1$^{XTR/XTR}$*-tamoxifen, n = 369 tumors; *KPT;Lkb1$^{XTR/XTR}$;FLPo-ER^{T2}*-vehicle, n = 375 tumors; *KPT;Lkb1$^{XTR/XTR}$;FLPo-ER^{T2}*-tamoxifen, n = 352 tumors. Red crossbars indicate the mean. One tissue section per mouse was analyzed. *P* values were calculated by two-sided unpaired t-test. VEH vehicle. TAM tamoxifen.

a

Genotype	Treatment	Number of mice	Number of tumors	Class
KT;Lkb1$^{XTR/XTR}$	Tamoxifen	4	10	Non-Restored
KT;Lkb1$^{XTR/XTR}$;FLPo-ER^{T2}	Vehicle	3	9	Non-Restored
KT;Lkb1$^{XTR/XTR}$;FLPo-ER^{T2}	Tamoxifen	3	10	Restored

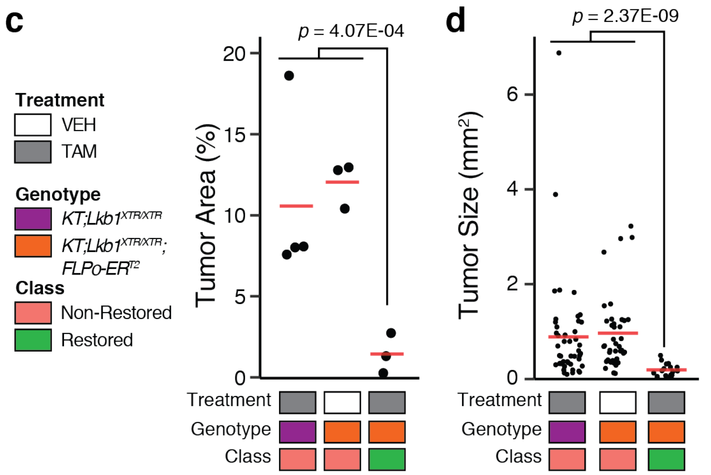

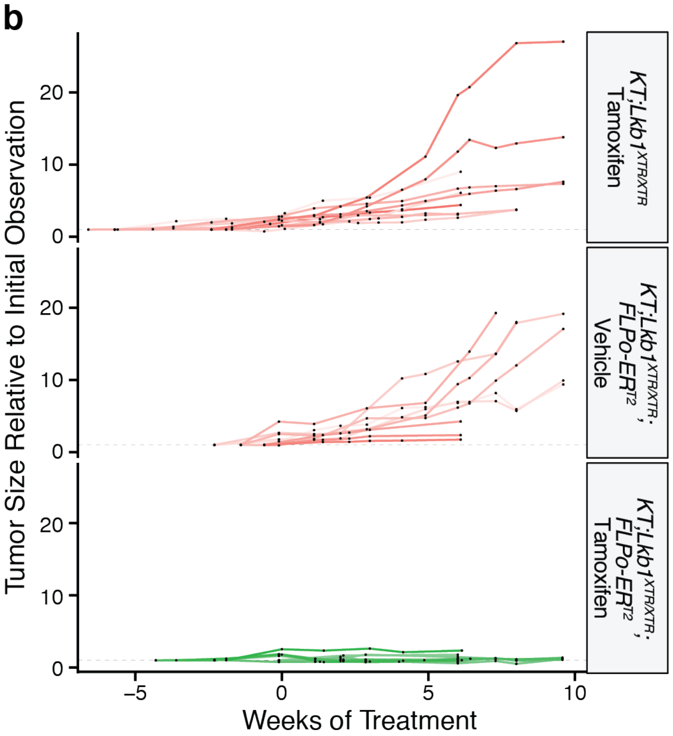

Supplementary Figure 6. Longitudinal analysis of tumor growth indicates that *Lkb1* restoration promotes tumor stasis.

a. Capturing the growth dynamics of *Lkb1*-restored and non-restored lung tumors via longitudinal μCT imaging. Summary of the mice enrolled, indicating mouse genotype, treatment, and the number of tumors within each that were longitudinally measured by μCT.

b. Tumor growth trajectories in terms of change in tumor volume relative to initial measurement (source data for aggregated metrics depicted in **Fig. 2b**). Each dot represents a single measurement of tumor size relative to initial observation at a given time point. Connected dots correspond to measurements of the same lesion at different time points. The number of tumors and mice analyzed for each cohort are summarized in **a**.

c, d. Measurements of tumor area (**c**) and size (**d**) as assessed by histology on tumor-bearing lungs collected upon termination of the μCT experiment. In **c**, each dot represents a mouse, while each dot in **d** corresponds to a tumor. Red crossbars indicate the mean. In **c**, *KT;Lkb1$^{XTR/XTR}$*-tamoxifen, $n = 4$ mice; *KT;Lkb1$^{XTR/XTR}$;FLPo-ER^{T2}*-vehicle, $n = 3$ mice; *KT;Lkb1$^{XTR/XTR}$;FLPo-ER^{T2}*-tamoxifen, $n = 3$ mice. In **d**, *KT;Lkb1$^{XTR/XTR}$*-tamoxifen, $n = 46$ tumors; *KT;Lkb1$^{XTR/XTR}$;FLPo-ER^{T2}*-vehicle, $n = 39$ tumors; *KT;Lkb1$^{XTR/XTR}$;FLPo-ER^{T2}*-tamoxifen, $n = 17$ tumors. *P* values were calculated by two-sided unpaired t-test. VEH vehicle. TAM tamoxifen.

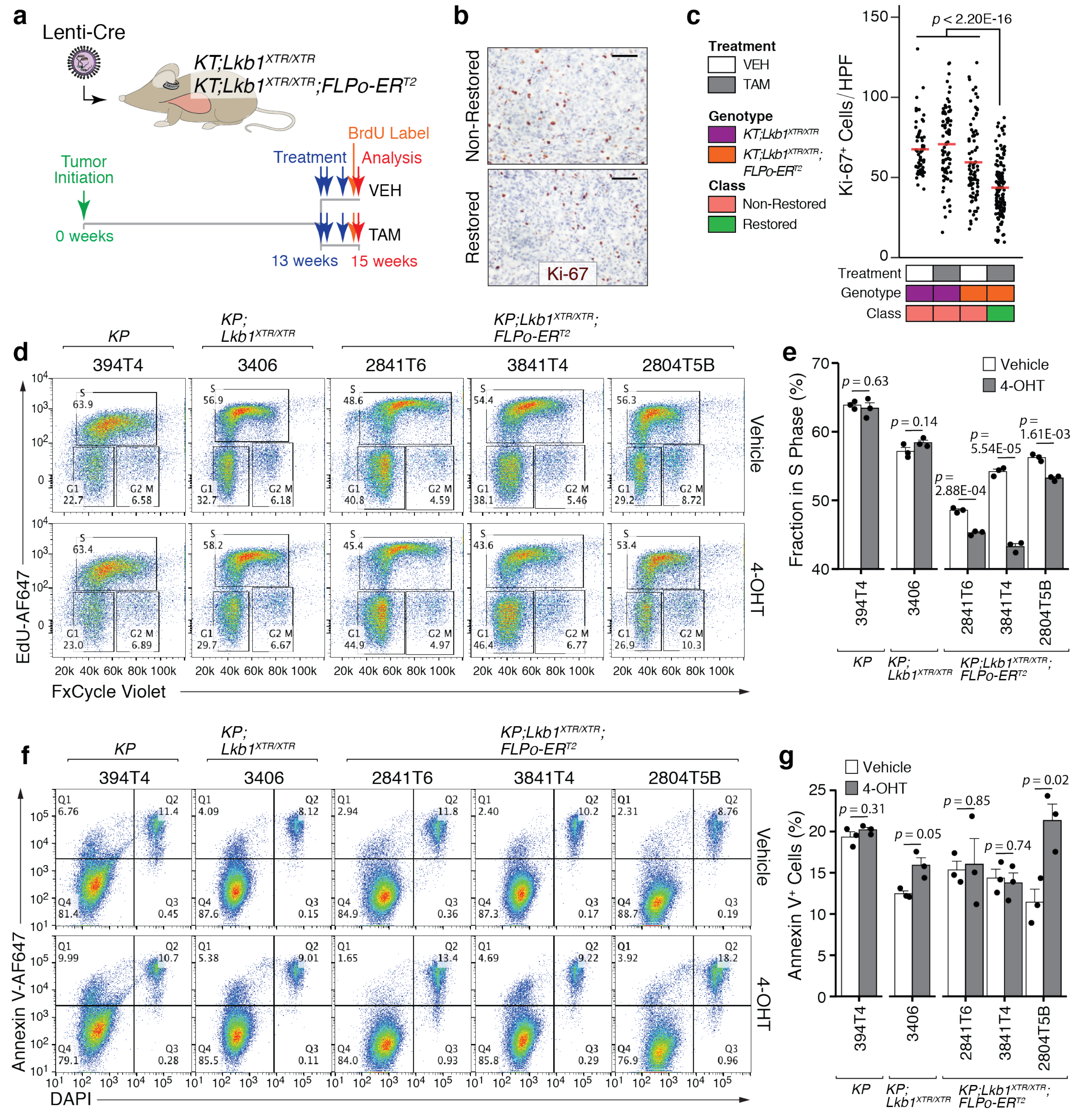

Supplementary Figure 7. *Lkb1* restoration suppresses proliferation and does not consistently induce cell death.

a. Examination of the acute response to *Lkb1* restoration in terms of changes in proliferation or cell death. Tumors were initiated in *KT;Lkb1$^{XTR/XTR}$*, and *KT;Lkb1$^{XTR/XTR}$;FLPo-ER^{T2}* mice via intratracheal delivery of Lenti-Cre. At 13 weeks post-initiation, tumor-bearing mice were treated for two weeks with vehicle or tamoxifen prior to analysis. Mice were administered BrdU intraperitoneally at 24 hours prior to harvesting tissues to assess proliferation. VEH vehicle. TAM tamoxifen.

b, c. Representative images and (**b**) and quantification (**c**) of proliferation as assessed by immunohistochemical staining for Ki-67. Each dot represents the frequency of positive cells detected within a single 20x field. Red crossbars indicate the mean. Scale bars = 100 μm. One tissue section per mouse was analyzed. *KT;Lkb1$^{XTR/XTR}$*-vehicle, *n* = 78 fields; *KT;Lkb1$^{XTR/XTR}$*-tamoxifen, *n* = 98 fields; *KT;Lkb1$^{XTR/XTR}$;FLPo-ER^{T2}*-vehicle, *n* = 114 fields; *KT;Lkb1$^{XTR/XTR}$;FLPo-ER^{T2}*-tamoxifen, *n* = 141 fields. *P* values were calculated by two-sided unpaired t-test. VEH vehicle. TAM tamoxifen.

d-g. Representative flow cytometry plots (**d, f**) and corresponding quantification for cell cycle analysis by EdU and FxCycle Violet staining (**d, e**) and cell death as assessed by Annexin V staining (**f, g**) in lung cancer cell lines derived from *KP* (*n* = 1 cell line), *KP;Lkb1$^{XTR/XTR}$* (non-restorable; *n* = 1 cell line) and *KP;Lkb1$^{XTR/XTR}$;FLPo-ER^{T2}* (restorable, *n* = 3 cell lines) mice. Annexin V^{+} cells were identified as those that fall within quadrants 1 and 2. Cells were analyzed 96 hours after initiation of treatment with ethanol vehicle (top) or 4-hydroxytamoxifen

(4-OHT; bottom) to induce nuclear FLPo-ER^{T2} activity and consequently the restoration of *Lkb1*. In **e** and **g**, each dot represents an individual technical replicate. Bars indicate the mean and error bars indicate standard error of the mean. *P* values were calculated by two-sided unpaired t-test. Representative of two independent experiments.

a

Genotype	Treatment	Number of mice	Number of tumors	Class
KT;Lkb1$^{XTR/XTR}$	Tamoxifen	5	22	Non-Restored
KT;Lkb1$^{XTR/XTR}$*;FLPo-ER*^{T2}	Tamoxifen	5	15	Restored

b

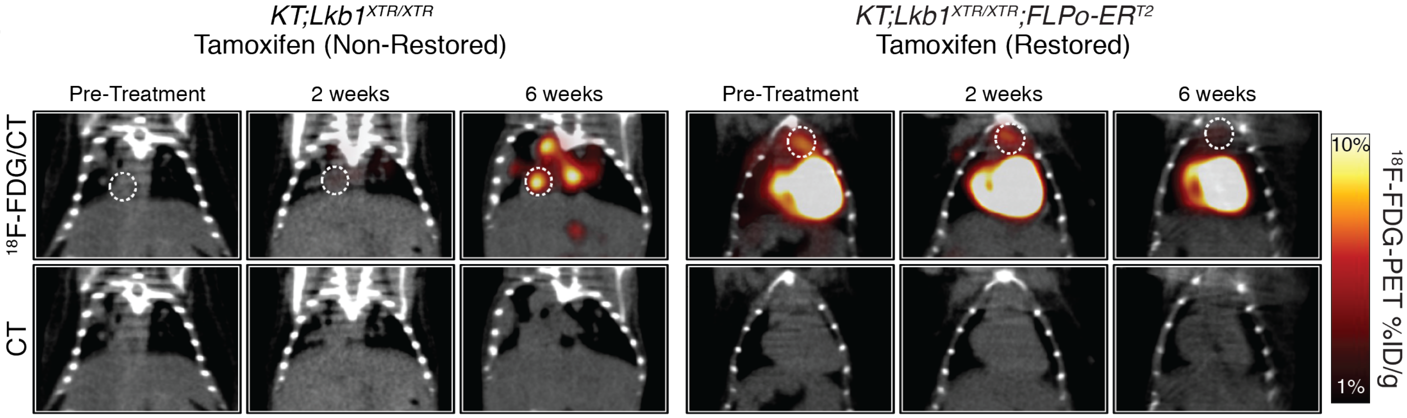

c

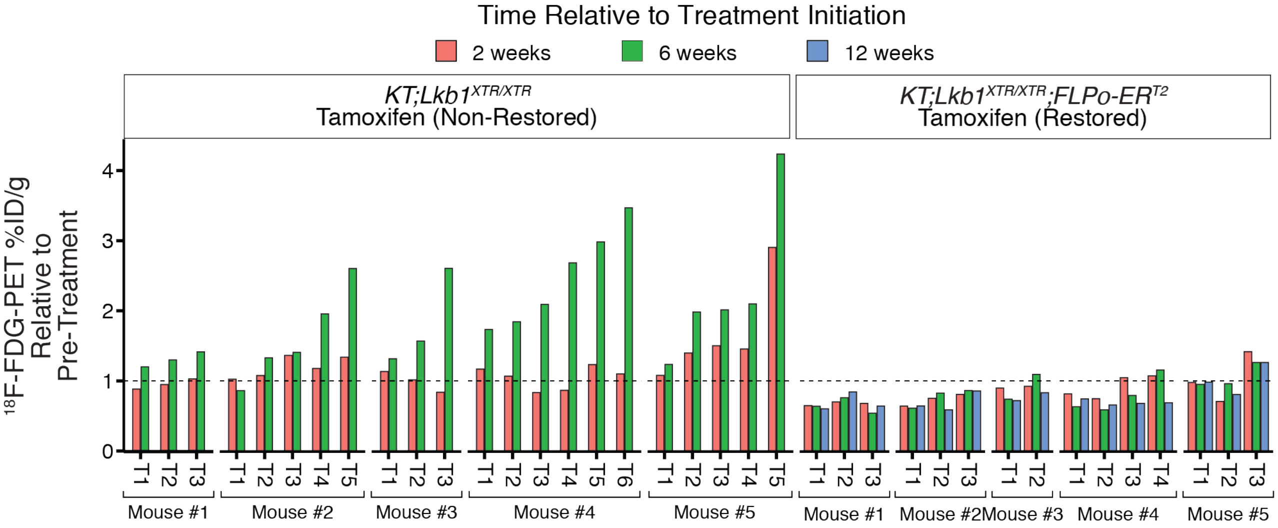

Supplementary Figure 8. *Lkb1* restoration abrogates the increase in glucose avidity that accompanies lung tumor progression.

a. Capturing longitudinal changes in glucose avidity in response to *Lkb1* restoration by serial ^{18}F-FDG-PET/CT imaging. Summary of the mice enrolled, indicating mouse genotype, treatment, and the number of tumors within each that were longitudinally measured. **b.** Representative μCT images (bottom) and overlaid ^{18}F-FDG signal (top) of tumor-bearing lungs from *Lkb1*-restored or non-restored mice at two days prior to treatment initiation as well as at two and six weeks after. Measured tumors are outlined by white, dashed lines. Signal intensity reported in terms of maximum percentage injected dose per gram (%ID/g).

c. Graphical summary of changes in ^{18}F-FDG uptake relative to initial measurement for individual *Lkb1* non-restored and restored tumors. Each bar represents a single measurement of change in ^{18}F-FDG uptake relative to initial observation for a given tumor at a given time point, with fill color corresponding to the time of measurement. Number of mice and tumors analyzed per cohort summarized in **a**. Tumors within the same animal are grouped along the x-axis. Source data for **Fig. 2h**.

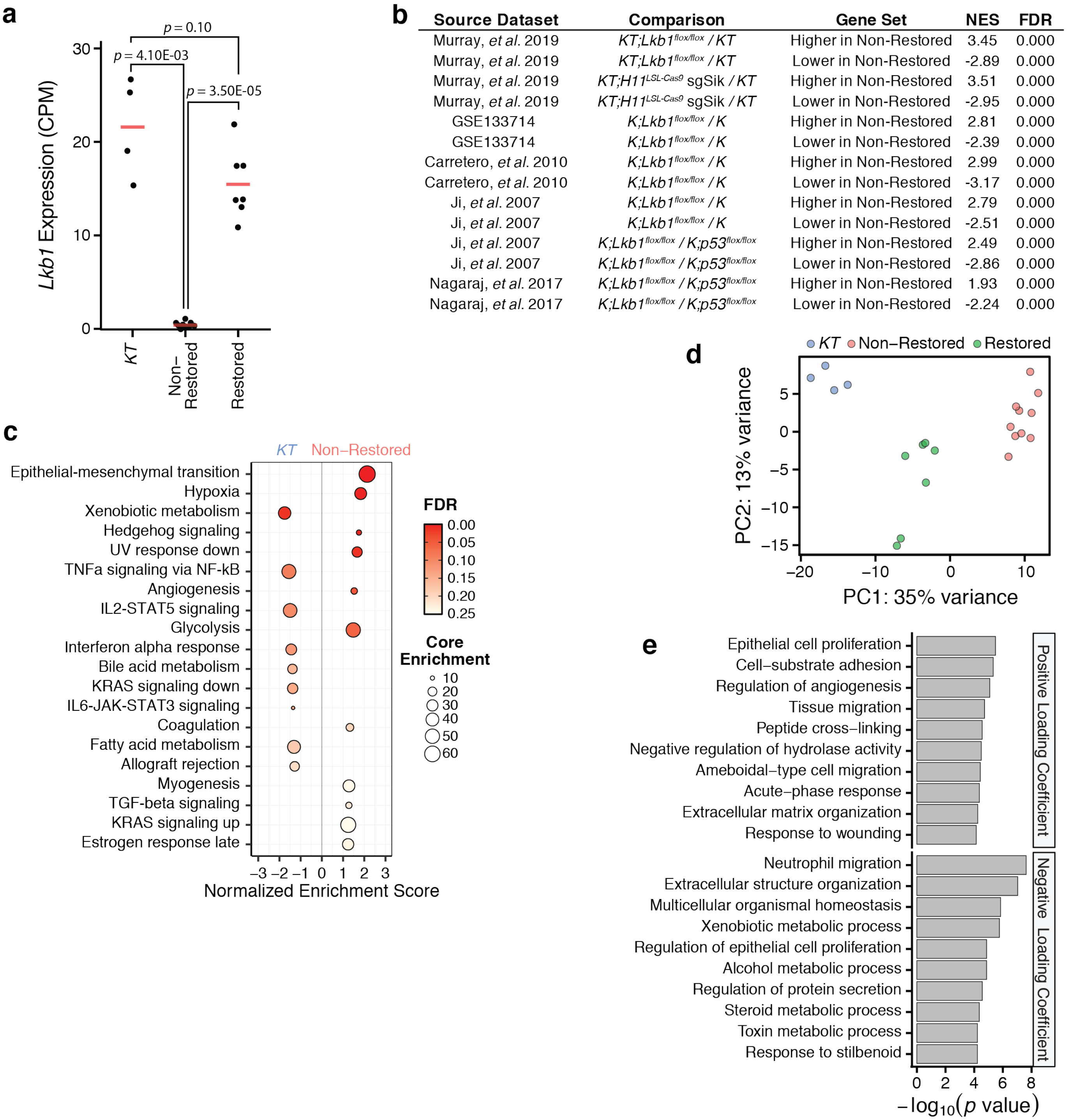

Source Dataset	Comparison	Gene Set	NES	FDR
Murray, *et al.* 2019	*KT;Lkb1*$^{flox/flox}$ / *KT*	Higher in Non-Restored	3.45	0.000
Murray, *et al.* 2019	*KT;Lkb1*$^{flox/flox}$ / *KT*	Lower in Non-Restored	-2.89	0.000
Murray, *et al.* 2019	*KT;H11*$^{LSL\text{-}Cas9}$ sgSik / *KT*	Higher in Non-Restored	3.51	0.000
Murray, *et al.* 2019	*KT;H11*$^{LSL\text{-}Cas9}$ sgSik / *KT*	Lower in Non-Restored	-2.95	0.000
GSE133714	*K;Lkb1*$^{flox/flox}$ / *K*	Higher in Non-Restored	2.81	0.000
GSE133714	*K;Lkb1*$^{flox/flox}$ / *K*	Lower in Non-Restored	-2.39	0.000
Carretero, *et al.* 2010	*K;Lkb1*$^{flox/flox}$ / *K*	Higher in Non-Restored	2.99	0.000
Carretero, *et al.* 2010	*K;Lkb1*$^{flox/flox}$ / *K*	Lower in Non-Restored	-3.17	0.000
Ji, *et al.* 2007	*K;Lkb1*$^{flox/flox}$ / *K*	Higher in Non-Restored	2.79	0.000
Ji, *et al.* 2007	*K;Lkb1*$^{flox/flox}$ / *K*	Lower in Non-Restored	-2.51	0.000
Ji, *et al.* 2007	*K;Lkb1*$^{flox/flox}$ / *K;p53*$^{flox/flox}$	Higher in Non-Restored	2.49	0.000
Ji, *et al.* 2007	*K;Lkb1*$^{flox/flox}$ / *K;p53*$^{flox/flox}$	Lower in Non-Restored	-2.86	0.000
Nagaraj, *et al.* 2017	*K;Lkb1*$^{flox/flox}$ / *K;p53*$^{flox/flox}$	Higher in Non-Restored	1.93	0.000
Nagaraj, *et al.* 2017	*K;Lkb1*$^{flox/flox}$ / *K;p53*$^{flox/flox}$	Lower in Non-Restored	-2.24	0.000

Supplementary Figure 9. Trapped *Lkb1*^{TR} conformation recapitulates transcriptional features of *Lkb1* inactivation.
a. *Lkb1* mRNA abundance in terms of counts per million (CPM) across *Lkb1* wild-type (*KT; n* = 4 tumors), non-restored (*n* = 10 tumors), and restored cohorts (*n* = 7 tumors). Red crossbars indicate the mean. *P* values were calculated by two-sided unpaired t-test.
b. GSEA on previously published gene expression data sets of mouse lung tumors that are *Lkb1* wild-type (*K* or *KT*), *Lkb1*-deficient (*K;Lkb1*$^{flox/flox}$ or *KT;Lkb1*$^{flox/flox}$), or Sik-targeted (*KT;H11*$^{LSL\text{-}Cas9}$ sgSik). Gene sets were defined by genes that are either significantly higher or lower in non-restored tumors relative to *Lkb1* wild-type (*KT*) tumors (absolute $\log_2$ Fold Change > 1 and FDR < 0.05).
c. GSEA comparing non-restored and *Lkb1* wild-type (*KT*) tumors using the Hallmarks module. Size of dots correspond to the number of core enrichment genes and the fill color reflects FDR.
d. Principal component analysis (PCA) of the transcriptional profiles of *Lkb1* wild-type (*KT*), non-restored, and restored tumors using the top 500 most variable genes. The proportion of variance captured by each principal component is indicated.
e. GO term Biological Process enrichment analysis of subsets of the top 500 most variable genes with either positive or negative loading coefficients with respect to the first principal component.

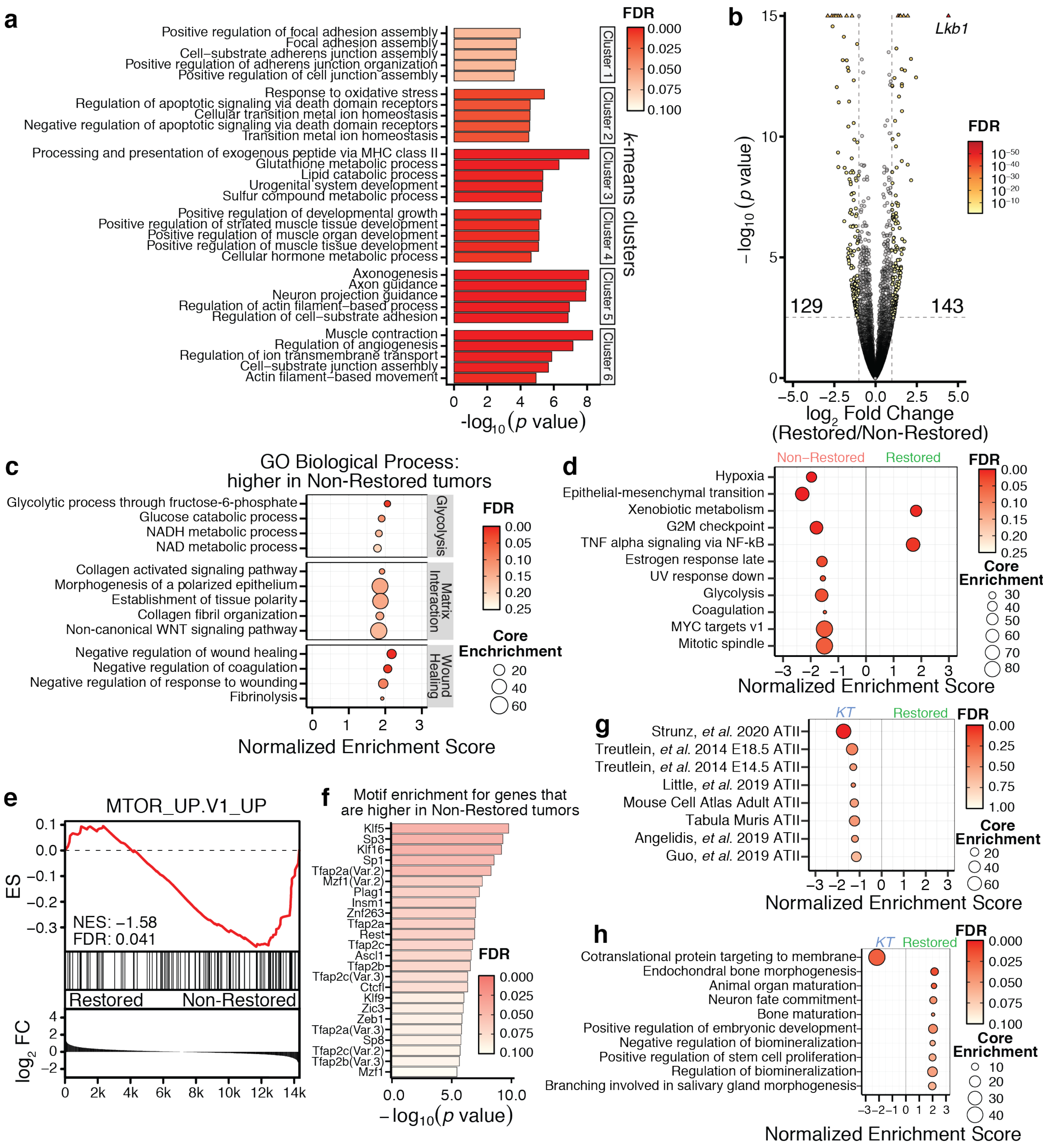

Supplementary Figure 10. Characterization of the acute transcriptional response to *Lkb1* restoration.
a. GO term Biological Process enrichment analysis for each *k*-means cluster defined in **Fig. 3c**. Includes the top five terms that were most significantly enriched within each cluster. Fill reflects the significance of the enrichment.
b. Volcano plot summarizing the gene expression changes between *Lkb1*-restored and non-restored tumors. The position of *Lkb1* is indicated. Dots corresponding to genes that were identified as differentially expressed (absolute $\log_2$ Fold Change > 1 and FDR < 0.05) have a fill color that reflects FDR. The number of differentially expressed genes in either direction is indicated.
c. GSEA comparing non-restored and restored tumors using the GO Biological Process module. Depicts the enrichment of groups of gene sets relating to glycolysis, extracellular matrix interactions, and wound healing among genes that are higher in non-restored tumors relative to restored tumors. Size of dots correspond to the number of core enrichment genes and the fill color reflects FDR.

d. GSEA comparing non-restored and restored tumors using the Hallmarks module. Size of dots correspond to the number of core enrichment genes and the fill color reflects FDR.

e. GSEA plot demonstrating the enrichment of mTOR signaling response genes among genes that are higher in non-restored tumors as compared to restored tumors. ES = Running Enrichment Score. NES = Normalized Enrichment Score. FC = fold change.

f. Transcription factor motif enrichment analysis on the putative promoters (-450 to +50 bp from transcription start site) of those genes that are higher in non-restored lung tumors relative to restored using the JASPAR 2018 collection of position frequency matrices.

g, h. GSEA comparing *Lkb1*-restored and *Lkb1* wild-type tumors using the signatures of ATII identity (**g**) and signatures from the GO Biological Process module (**h**). Size of dots correspond to the number of core enrichment genes and the fill color reflects FDR.

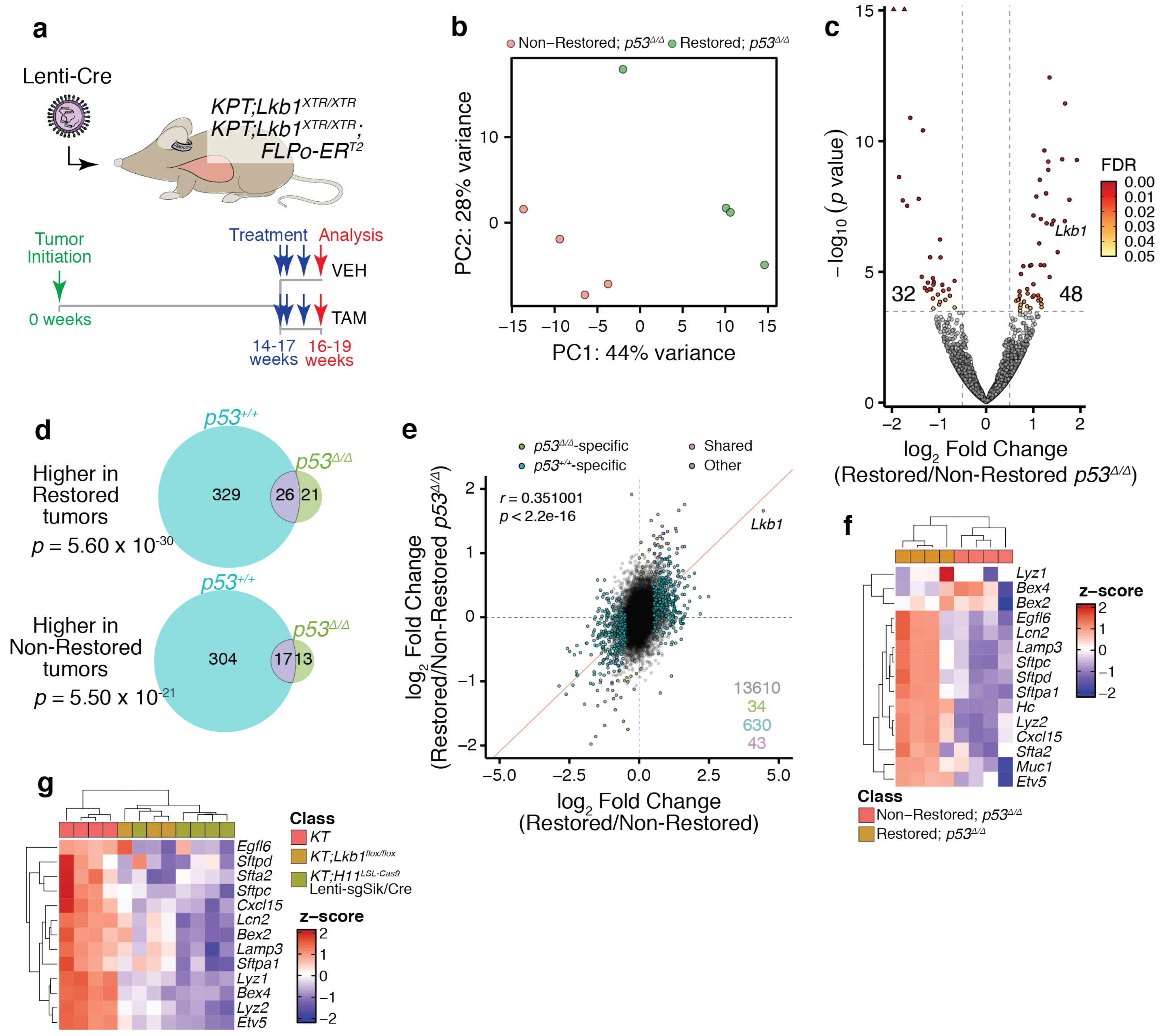

Supplementary Figure 11. *Lkb1* reactivation in the context of *p53* inactivation elicits gene expression changes that overlap with those identified in the *p53* wild-type setting.

a. Capturing the acute transcriptional response to *Lkb1* restoration in the absence of p53. Tumors were initiated in *KPT;Lkb1*$^{XTR/XTR}$, and *KPT;Lkb1*$^{XTR/XTR}$*;FLPo-ER*^{T2} mice via intratracheal delivery of Lenti-Cre. After 14-17 weeks, mice were treated with vehicle or tamoxifen for two weeks prior to isolation of tdTomato^{positive} neoplastic cells by FACS for RNA-seq. *KPT;Lkb1*$^{XTR/XTR}$-tamoxifen, n = 2 tumors; *KPT;Lkb1*$^{XTR/XTR}$*;FLPo-ER*^{T2}-vehicle, n = 2 tumors; *KPT;Lkb1*$^{XTR/XTR}$*;FLPo-ER*^{T2}-tamoxifen, n = 4 tumors. VEH vehicle. TAM tamoxifen.
b. Principal component analysis of the transcriptional profiles of non-restored, and restored tumors in which p53 has been concomitantly inactivated using the top 500 most variable genes. The proportion of variance captured by each principal component is indicated.
c. Transcriptional comparison of *Lkb1*-restored and non-restored tumors in which *Trp53* has been concomitantly inactivated. The position of *Lkb1* is indicated. Dots corresponding to genes that were identified as differentially expressed (absolute $\log_2$ Fold Change > 0.5 and FDR < 0.05) have a fill color that reflects FDR. The number of differentially expressed genes in either direction is indicated.
d. Direct comparison of differentially expressed genes (absolute $\log_2$ Fold Change > 0.5 and FDR < 0.05) between *Trp53* wild-type and *Trp53*-deficient settings. Venn diagrams depict the overlap of genes that are either higher in restored tumors (top) or higher in non-restored tumors (bottom). *P* values from hypergeometric tests are indicated.
e. Scatter plot to assess concordance in gene expression changes between restored and non-restored tumors across *Trp53* wild-type and *Trp53*-deficient settings. Deming regression line shown in red. Correlation coefficient and corresponding *p* value were determined by two-sided Pearson correlation test (top left). The number of differentially expressed genes (absolute $\log_2$ Fold Change > 0.5 and FDR < 0.05) that are shared between or unique to either *Trp53* wild-type or *Trp53*-deficient settings are listed (bottom right).

f, g. Heatmap displaying the expression of markers associated with alveolar type II epithelial cell identity across *Lkb1* non-restored and restored tumors in which *Trp53* has been concomitantly inactivated (**f**), in addition to *Lkb1* wild-type (*KT*) and *Lkb1*-deficient tumors (*KT*; *Lkb1$^{flox/flox}$*), as well as Sik-targeted (*KT;H11$^{LSL\text{-}Cas9}$* Lenti-sgSik/Cre) tumors from a previously published RNA-seq dataset[36].

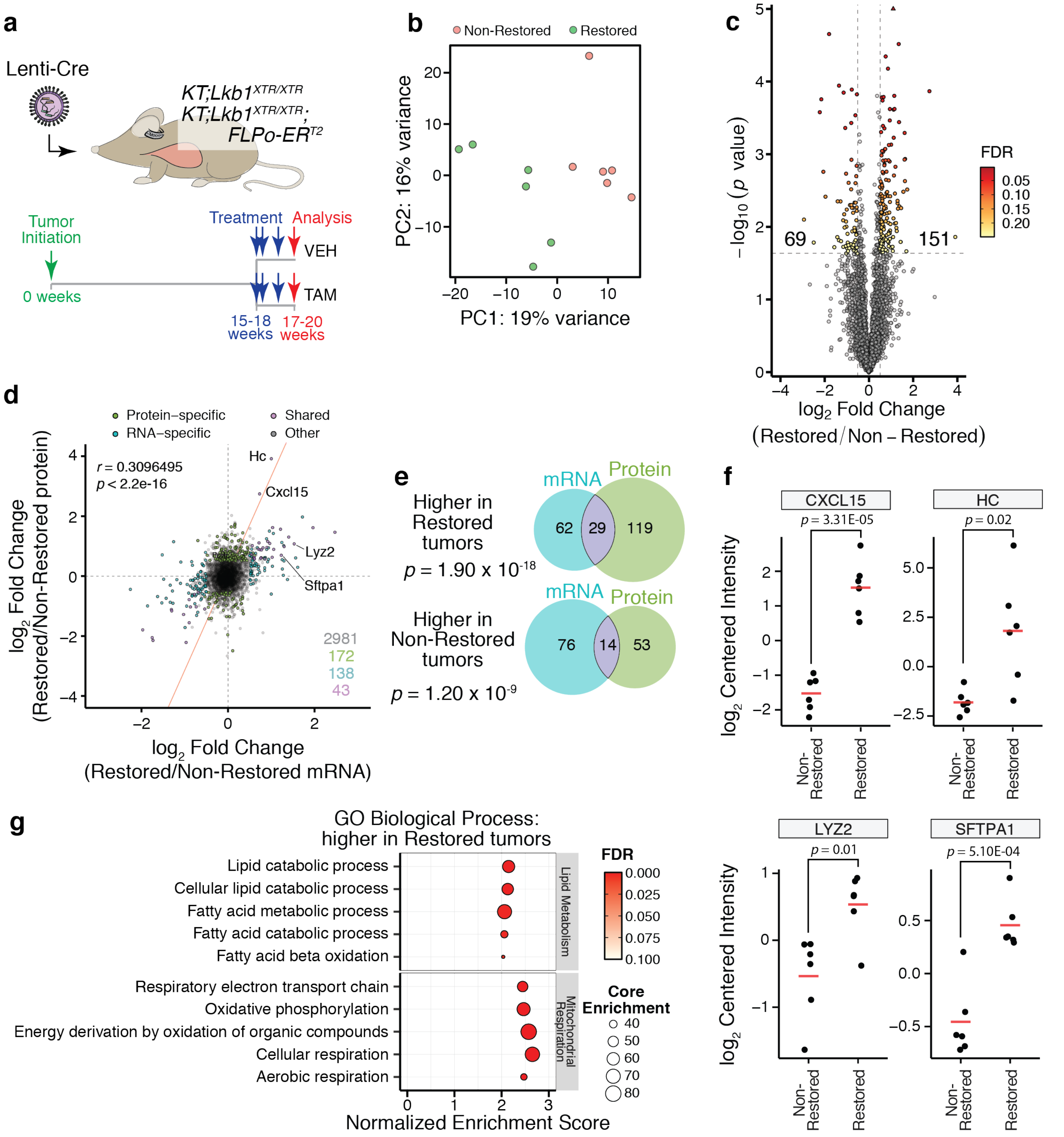

Supplementary Figure 12. Proteomic profiling of the acute response to *Lkb1* restoration.

a. Characterizing the response to *Lkb1* restoration at the protein level. Tumors were initiated in *KT;Lkb1*$^{XTR/XTR}$, and *KT;Lkb1*$^{XTR/XTR}$*;FLPo-ER*^{T2} mice via intratracheal delivery of Lenti-Cre. At 15-18 weeks post-initiation, mice were treated with vehicle or tamoxifen for two weeks prior to isolation of tdTomato^{positive} neoplastic cells by FACS for proteomic profiling. *KT;Lkb1*$^{XTR/XTR}$-vehicle, $n = 1$ tumor; *KT;Lkb1*$^{XTR/XTR}$-tamoxifen, $n = 1$ tumor; *KT;Lkb1*$^{XTR/XTR}$*;FLPo-ER*^{T2}-vehicle, $n = 4$ tumors; *KT;Lkb1*$^{XTR/XTR}$*;FLPo-ER*^{T2}-tamoxifen, $n = 6$ tumors. VEH vehicle. TAM tamoxifen.

b. Principal component analysis of *Lkb1*-restored and non-restored tumors using the top 500 most variable proteins. The proportion of variance captured by each principal component is indicated.

c. Comparison of protein abundance between *Lkb1*-restored and non-restored tumors. Dots corresponding to differentially abundant proteins (absolute $\log_2$ Fold Change > 0.5 and FDR < 0.25) have a fill color that reflects FDR. The number of differentially abundant proteins in either direction is indicated.

d. Scatter plot to assess concordance in terms of changes in protein and RNA abundance. Deming regression line shown in red. Correlation coefficient and corresponding *p* value were determined by two-sided Pearson correlation test (top left). The number of differentially expressed genes (absolute $\log_2$ Fold Change > 0.5 and FDR cutoffs of 0.05 and 0.25 for RNA and protein, respectively) that are identified uniquely at the protein or RNA levels or both (bottom right).
e. Venn diagrams depicting the overlap of genes that are either higher in restored tumors (top) or higher in non-restored tumors (bottom) at the RNA and protein levels (absolute $\log_2$ Fold Change > 0.5 and FDR cutoffs of 0.05 and 0.25 for RNA and protein, respectively). *P* values from hypergeometric tests are indicated.
f. Log_2 centered intensities for proteins associated with alveolar type II cells in *Lkb1*-restored (n = 6 tumors) and non-restored tumors (n = 6 tumors). Crossbars indicate the mean. *P* values were calculated by two-sided unpaired t-test.
g. GSEA comparing *Lkb1*-restored and non-restored tumors using GO Biological Process. Lipid metabolism (top) and mitochondrial respiration (bottom) gene sets enriched among proteins that are higher within restored tumors. Size of dots correspond to the number of core enrichment genes and the fill color reflects FDR.

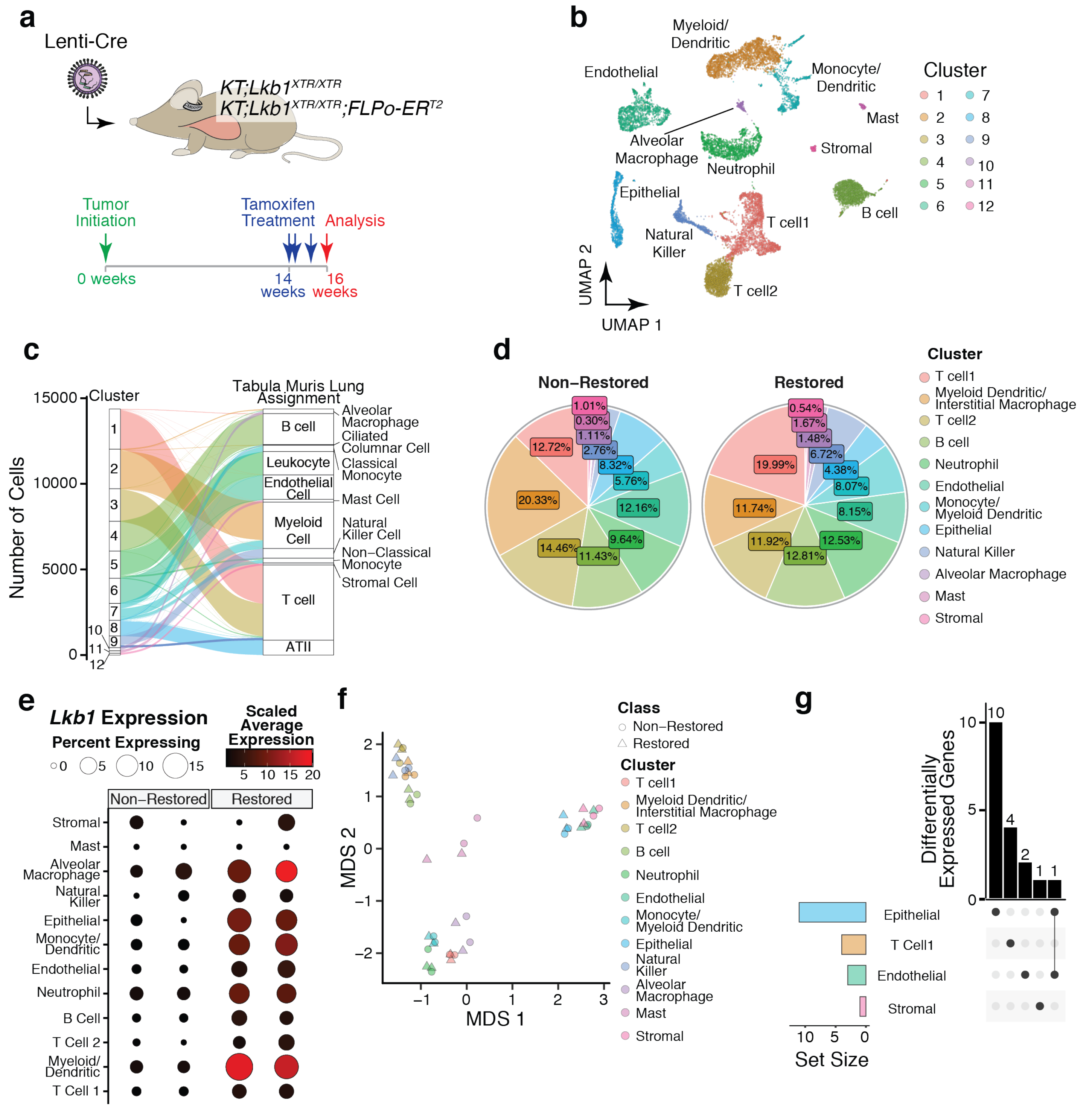

Supplementary Figure 13. The majority of early transcriptional changes induced by *Lkb1* restoration are confined to the epithelial compartment.

a. Assessment of the impact of *Lkb1* restoration on the abundance and/or state of the various cell populations within established lung tumors. Tumors were initiated in $KT;Lkb1^{XTR/XTR}$, and $KT;Lkb1^{XTR/XTR};FLPo\text{-}ER^{T2}$ mice via intratracheal delivery of Lenti-Cre. At 16 weeks post-initiation, following two weeks of treatment with tamoxifen, lung tumors were harvested and dissociated prior to isolation of viable cells by FACS for single-cell RNA-seq. $N = 2$ mice per genotype.

b. Single-cell transcriptomic profiles of total cells harvested from *Lkb1*-restored and non-restored tumors (two replicates each) embedded in UMAP space. Cells are colored according to the 11 clusters defined by the Louvain algorithm. Each cluster is labeled according to the predicted cell type identities using the Tabula Muris adult mouse lung dataset as a reference[71].

c. Sankey plot depicting the proportion of cells within each cluster that have been assigned to a cellular identity defined within the Tabula Muris adult mouse lung dataset. Ribbon color reflects Louvain cluster assignments.

d. Pie charts depicting the relative abundance of each cell type identified within *Lkb1*-restored and non-restored tumors. Sector fill reflects Louvain cluster assignments.

e. Dot plot showing *Lkb1* expression across cell types. Predicted cluster identities are listed to the left. Fill indicates average expression and dot size reflects the proportion of cells within a given cell-type specific cluster that express *Lkb1.*

f. Multi-dimensional scaling plot of single cell data at the pseudobulk level. Each symbol corresponds to a cell type-instance that was collapsed into a single sample. Symbol shape reflects whether the cell subpopulation was derived from either an *Lkb1*-restored or non-restored tumor. Symbol fill reflects Louvain cluster assignments, and the corresponding predicted cell identities are listed to the right.

g. UpSet plot summarizing the number of unique and shared differentially expressed genes that were identified within each cellular compartment between *Lkb1*-restored and non-restored tumors. The vertical bar plot depicts the number of differentially expressed genes that are uniquely identified within each cell type or cell-type intersection (number listed at the top of each bar). Horizontal bar plot (bottom) illustrating the number of differentially expressed genes identified within each cellular compartment.

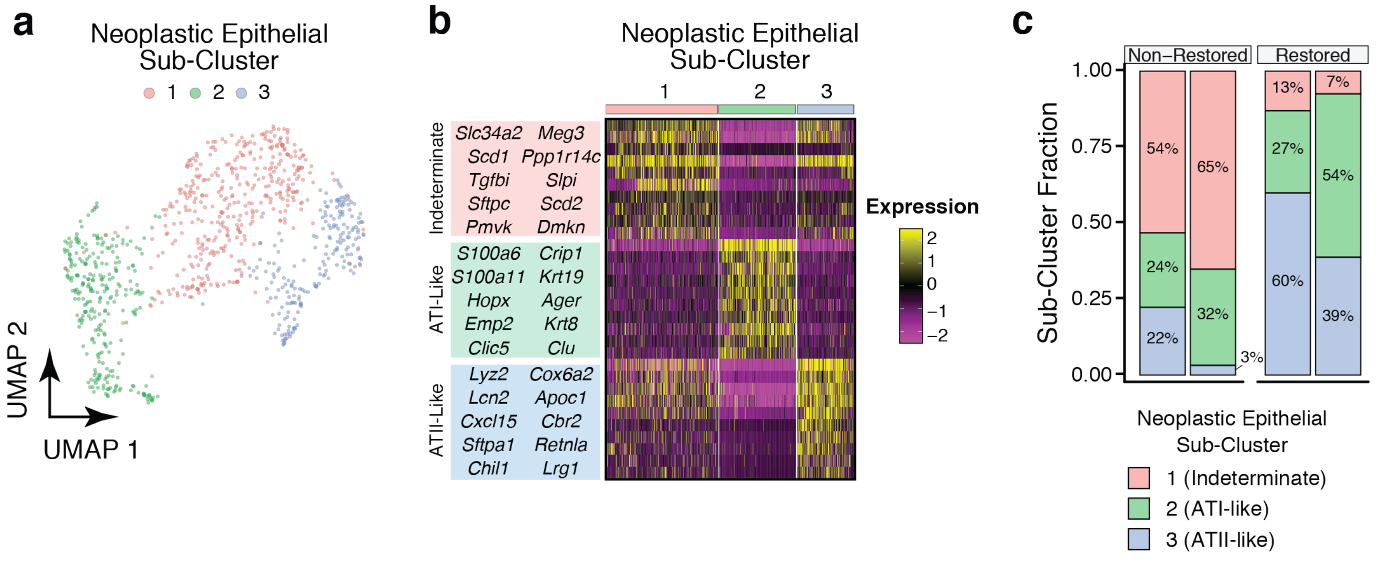

Supplementary Figure 14. *Lkb1* restoration promotes a cell-state transition within the alveolar epithelial compartment.

a. Sub-clustering of the epithelial compartment of restored and non-restored tumors plotted in UMAP space. Cells are colored according to the three sub-clusters defined by the Louvain algorithm.

b. Heatmap summarizing the top 10 markers that define each of the epithelial sub-clusters. The predicted cell type identities on the basis of these markers are listed to the left. Louvain cluster assignments are indicated by the bars lining the top of the heatmap.

c. Relative proportion of each epithelial subpopulation within *Lkb1*-restored and non-restored tumors (two replicates each). Fill reflects Louvain cluster assignments. Illustrates shift in tumor composition from indeterminate to ATII-like state between non-restored and restored tumors. Columns correspond to individual tumor samples.

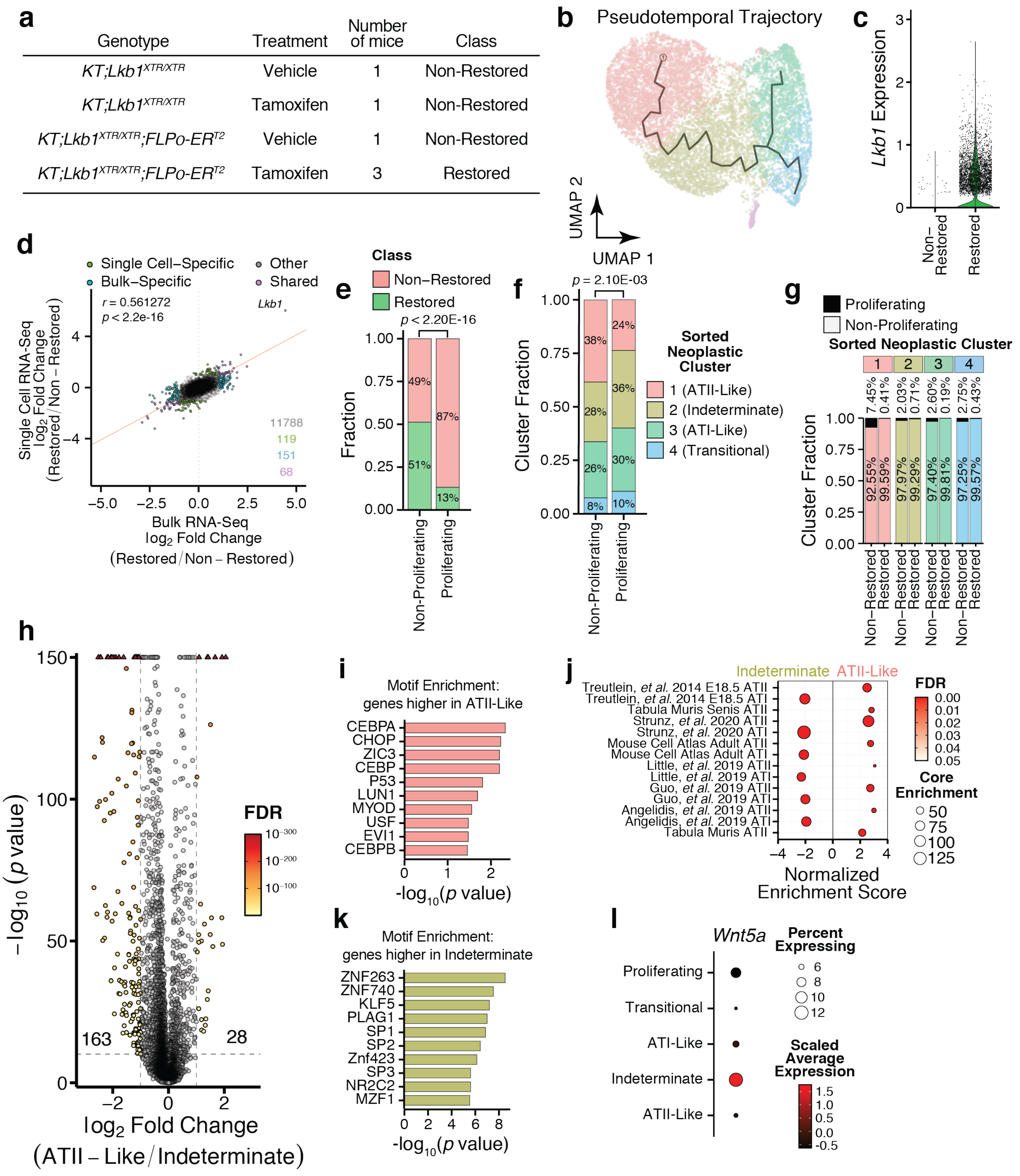

Supplementary Figure 15. Enhanced resolution within the neoplastic compartment reveals that LKB1 drives alveolar type II differentiation.

a. Table summarizing the mice from which tdTomato^{positive} neoplastic cells were isolated for single-cell RNA-seq.

b. Trajectory inference on sorted neoplastic cell clusters. Cell fill corresponds to Louvain cluster assignments.

c. *Lkb1* expression across single cells derived from either *Lkb1*-restored or non-restored tumors.

d. Gene expression changes induced by *Lkb1* restoration across bulk and single-cell experiments. Deming regression line shown in red. Correlation coefficient and corresponding *p* value were determined by two-sided Pearson correlation test (top left). The number of differentially expressed genes (absolute $\log_2$ Fold Change > 1 and FDR < 0.05) that are shared or unique to bulk or single-cell RNA-seq settings are listed (bottom right).

e. Proportion of cells derived from either *Lkb1*-restored or non-restored tumors within the non-proliferative and actively proliferating subpopulations. Significance corresponds to two-sided Chi-square test.

f. Proportion of cells assigned to each Louvain cluster from either the non-proliferative or actively proliferating fractions. The actively proliferating subpopulation was deconvoluted via regression of variation in genes relating to S and G2M phases. Significance corresponds to two-sided Chi-square test.

g. Proportion of proliferative cells within each Louvain cluster in *Lkb1*-restored and non-restored tumors. Bar fill reflects proliferative status and Louvain cluster assignment.

h. Transcriptional comparison of ATII-like and indeterminate clusters. Dots corresponding to differentially expressed genes (absolute $\log_2$ Fold Change > 1 and FDR < 0.05) have a fill color that reflects FDR. The number of differentially expressed genes in either direction is indicated.

i, k. Transcription factor motif enrichment on genes (-450 to +50 bp from transcription start site) that are higher in ATII-like cells (**I**) or indeterminate cells (**k**).

j. GSEA comparing ATII-like and indeterminate clusters at the pseudobulk level using signatures of ATI and ATII identity. Size of dots correspond to the number of core enrichment genes and the fill color reflects FDR. Shown are gene sets that are enriched among the genes that are higher in the indeterminate cluster.

l. *Wnt5a* expression across neoplastic cell states. Predicted cluster identities are listed to the left. Fill indicates average expression and dot size reflects the proportion of cells within a given cluster that express *Wnt5a*.

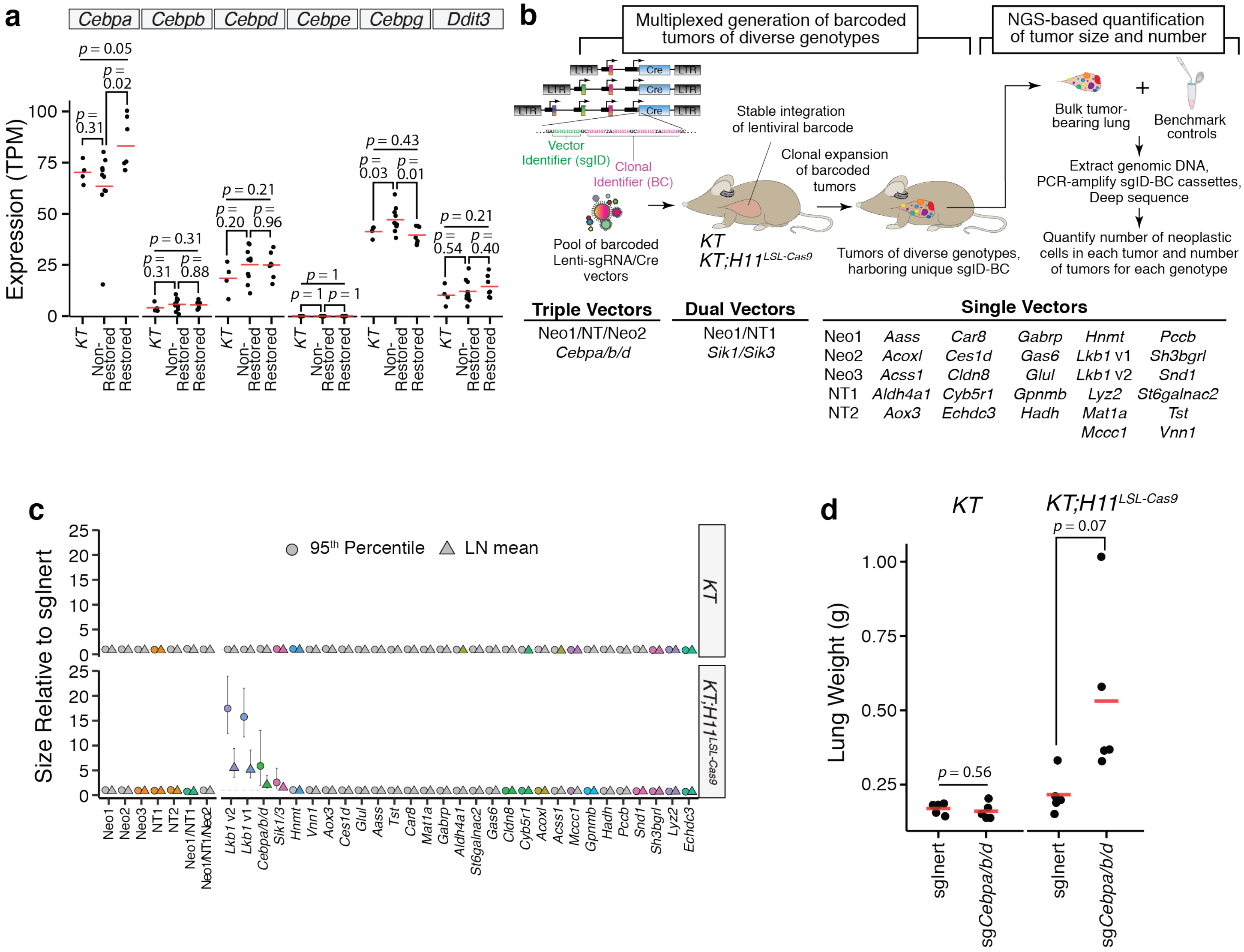

Supplementary Figure 16. C/EBP transcription factors are suppressors of oncogenic KRAS-driven lung tumor growth.

a. Transcript abundance for C/EBP factors across *Lkb1* wild-type (*KT; n* = 4 tumors), non-restored (*n* = 10 tumors), and restored cohorts (*n* = 7 tumors) in transcripts per million (TPM). Red crossbars indicate the mean. *P* values were calculated by two-sided unpaired t-test.
b. Assessing the tumor-suppressive capacity of various candidate genes in a multiplexed format. Lenti-sgRNA/Cre vectors are modified with two-component barcodes to tag the genomes of transduced cells with unique clonal identifiers (BC) and vector-specific identifiers (sgID) that correspond to the sgRNA encoded within the vector. Barcoded lentiviruses are delivered to the lung in a pooled format, resulting in the formation of uniquely barcoded, genotypically diverse lung tumors. The size and genotype of each individual clone can then be recovered by PCR-amplification and deep sequencing of lentiviral barcodes from bulk lung genomic DNA. The sgRNA pool targeted a subset of LKB1-dependent genes as identified by RNA-seq as well as a subset of C/EBP factors as indicated. The pool also comprised five inert sgRNA and two sgRNAs targeting *Lkb1* in addition to a vector targeting both *Sik1* and *Sik3*. Tumors were initiated in *KT* and *KT;H11^{LSL-Cas9}* mice and allowed to develop for 14 weeks prior to harvesting for Tuba-seq analysis. Triple vectors encoded sgRNAs as a tandem array driven by bovine U6, mouse U6, and human U6 promoters. Dual vectors encoded sgRNAs as a tandem array driven mouse and human U6 promoters. Single vectors encoded human U6-sgRNA cassettes.
c. Graphical summary of tumor size at the 95th percentile (circle) and log-normal mean (triangle) for each sgRNA relative to the size of tumors initiated with inert sgRNAs. Data are shown for both Cas9-deficient (*KT*; top) or proficient (*KT;H11^{LSL-Cas9}*) settings. Fill color reflects whether tumor size is significantly different, gray fill indicates no significant difference. Error bars indicate 95% confidence intervals centered on the mean of relative tumor size.
d. Lung weights for *KT* and *KT;H11^{LSL-Cas9}* mice transduced with either Lenti-sgInert/Cre or Lenti-sg*Cebpa/b/d*/Cre. Each dot corresponds to a mouse. *N* = 5 mice per genotype-virus cohort. Red crossbars indicate the mean. *P* values were calculated by two-sided unpaired t-test.

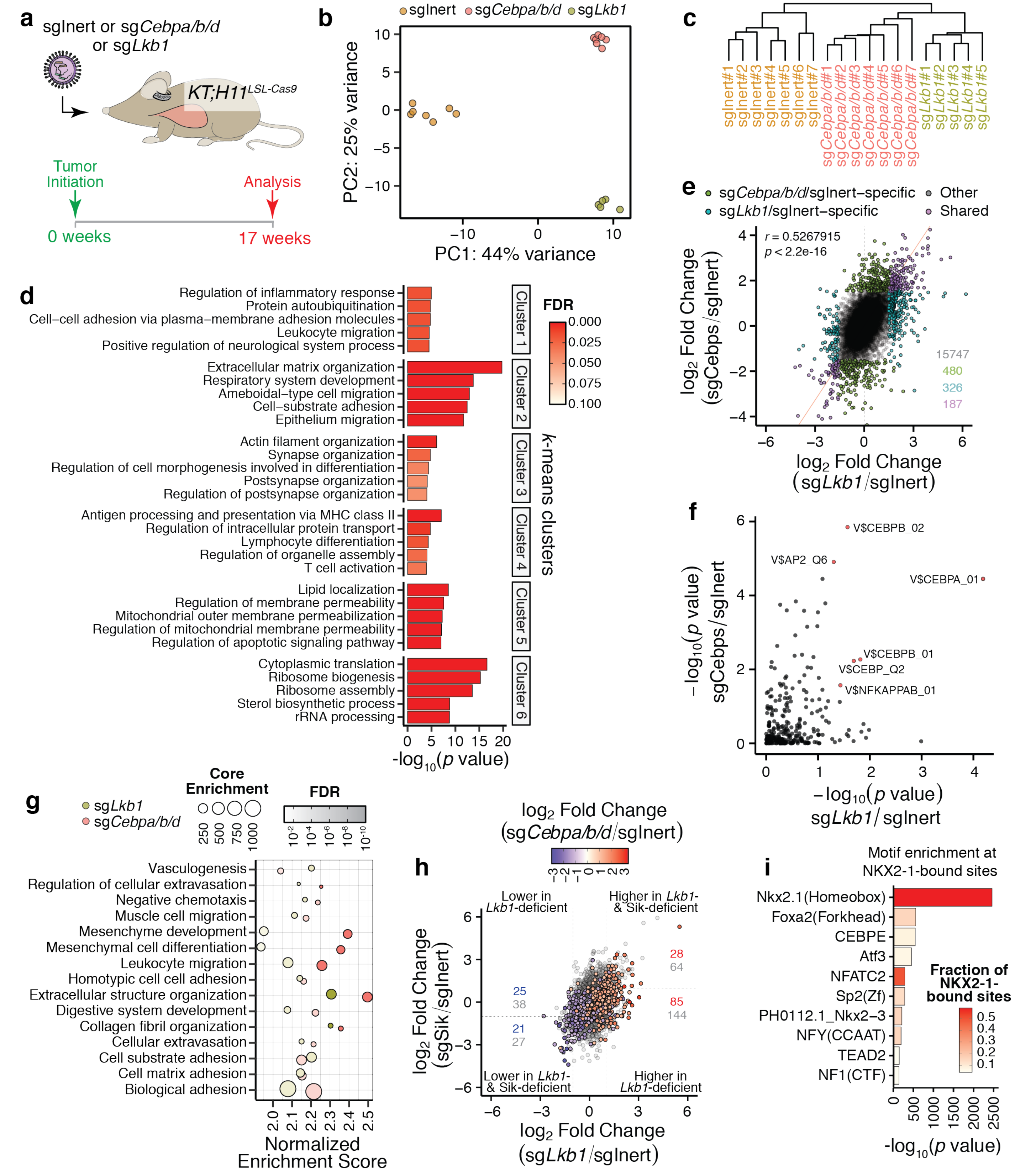

Supplementary Figure 17. Targeting of C/ebp factors recapitulates a subset of transcriptional changes that arise from *Lkb1* inactivation.

a. Transcriptional comparison of *Lkb1*- and C/ebp-targeted tumors. Lung tumors were initiated in $KT;H11^{LSL\text{-}Cas9}$ mice with Lenti-sg*Lkb1*/Cre (sg*Lkb1*, *n* = 5 tumors), Lenti-sg*Cebpa/b/d*/Cre (sg*Cebpa*/sg*Cebpb*/sg*Cebpd*; sg*Cebpa/b/d*, *n* = 7 tumors), or Lenti-sgInert/Cre (sgNeo1/sgNT1/sgNeo2; sgInert, *n* = 7 tumors). $tdTomato^{positive}$ neoplastic cells were isolated by FACS after 17 weeks for RNA-seq.

b, c. PCA (top 500 most variable genes) and hierarchical clustering by Euclidean distance.

d. GO Biological Process enrichment analysis for each *k*-means cluster defined in **Fig. 6a**.

e. Gene expression changes in *Lkb1*- and C/ebp-targeted tumors relative to tumors driven by oncogenic KRAS alone (sgInert). Deming regression line shown in red. Correlation coefficient and corresponding *p* value were determined by two-sided Pearson correlation test (top left). The number of differentially expressed genes (absolute log_2 Fold Change > 1 and FDR < 0.05) that are shared or unique to the *Lkb1*- or C/ebp-targeted settings are listed (bottom right).

f. Transcription factor motif enrichment on genes (-450 to +50 bp from transcription start site, TRANSFAC) that are lower in *Lkb1*- or C/ebp-targeted tumors relative to tumors driven by oncogenic KRAS alone (sgInert; log_2 Fold Change < -1 and FDR < 0.05). Shared enriched motifs (FDR < 0.05) are filled red and labeled.

g. GO Biological Process enrichment analysis on *Lkb1*- and C/ebp-targeted tumors (FDR < 0.05). Dot size reflects the size of the gene set overlap and fill corresponds to FDR.

h. Gene expression changes in *Lkb1*- and Sik-targeted tumors relative to tumors driven by oncogenic KRAS alone (sgInert). Dot fill reflects the log_2 fold changes between sg*Cebpa/b/d*- and sgInert-initiated tumors. Numbers within the quadrants correspond to the number of genes that significantly change upon *Lkb1* inactivation and are partitioned along the y-axis according to whether the genes also change significantly upon Sik targeting[36]. Colored numbers correspond to the number of genes that change concordantly in the C/ebp-targeted context, whereas those that do not are colored gray.

i. Motif enrichment using previously published ChIP-seq dataset that identified NKX2-1-bound sites within neoplastic cells isolated from oncogenic KRAS-driven mouse lung tumors[85]. Bar fill corresponds to the proportion of NKX2-1-bound sites that harbor a given transcription factor motif.

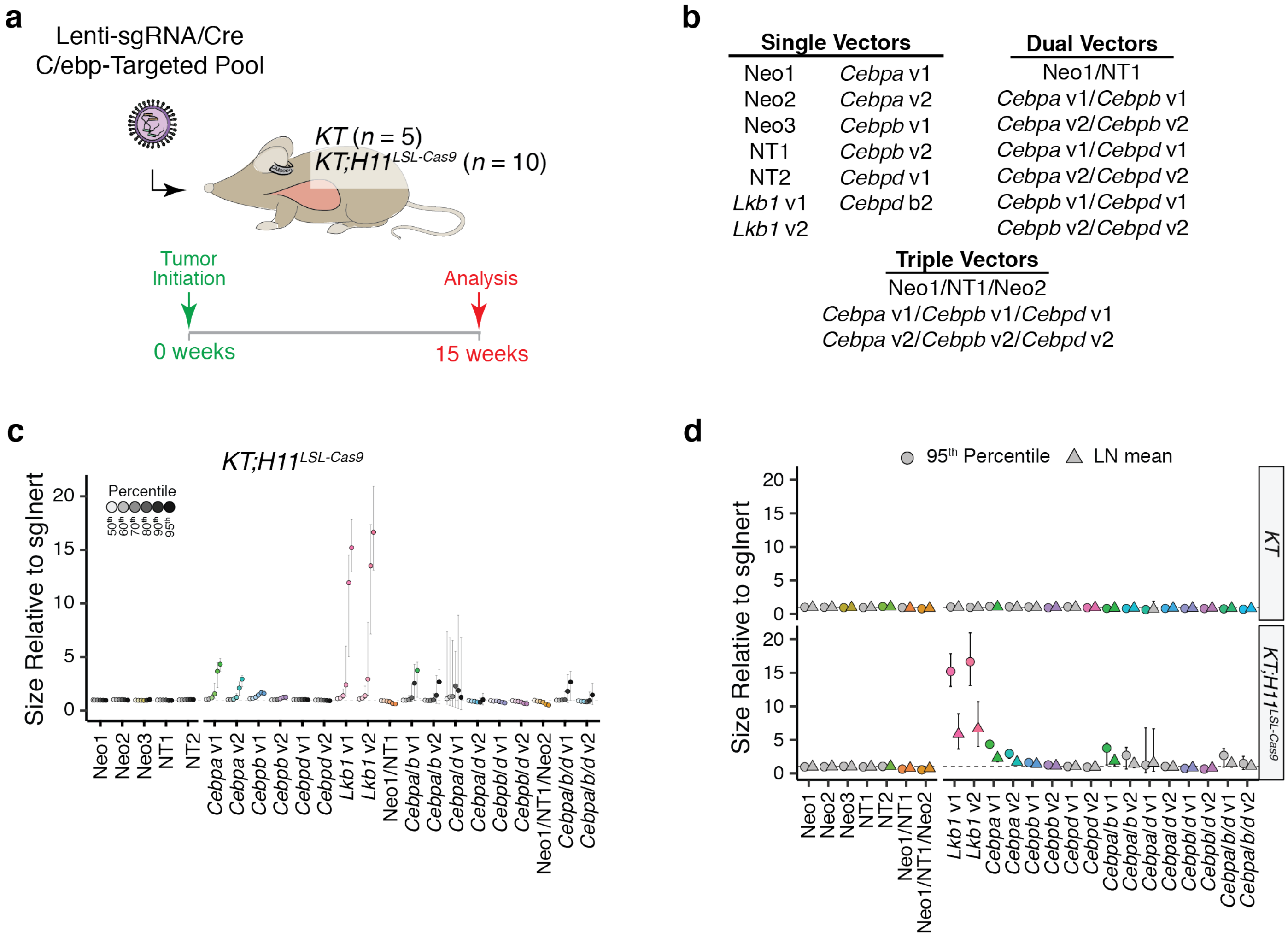

Supplementary Figure 18. C/EBPα is the dominant tumor-suppressive paralog among the C/EBP transcription factors.

a. Outline of secondary Tuba-seq screen to identify the tumor-suppressive paralogs among the previously targeted C/EBP members (*Cebpa*, *Cebpb*, *Cebpd*). The Lenti-sgRNA/Cre vectors incorporated within the lentiviral pool are listed in **b**. Tumors were initiated in *KT* and *KT;H11*$^{LSL\text{-}Cas9}$ mice via intratracheal delivery. At 15 weeks post-initiation, tumor-bearing lungs were harvested for Tuba-seq analysis to recover the tumor size distributions for each Lenti-sgRNA/Cre vector.

b. Outline of the vectors that were included in the pool for the secondary Tuba-seq screen. This pool encompassed a series of vectors encoding sgRNAs to target *Cebpa*, *Cebpb*, and *Cebpd* simultaneously, in pairwise combinations, and individually with two sets of independent sgRNAs for each target. Triple vectors encoded sgRNAs as a tandem array driven by bovine U6, mouse U6, and human U6 promoters. Dual vectors encoded sgRNAs as a tandem array driven mouse and human U6 promoters. Single vectors encoded human U6-sgRNA cassettes.

c. Summary of results from Tuba-seq experiment to interrogate the tumor-suppressive capacity of C/EBP factors. Percentile plot depicting tumor size at several percentiles relative to the distribution of tumors initiated with Lenti-sgRNA/Cre vectors encoding inert sgRNAs (sgNeo1, sgNeo2, sgNeo3, sgNT1, sgNT2). Each sgRNA species is assigned a distinct fill color, and fill saturation varies by percentile. Colored fill indicates that tumor size at a given percentile is significantly different from inert sgRNAs, while grayscale fill indicates no significant difference. Error bars indicate 95% confidence intervals centered on the mean relative tumor size.

d. Summary of the 95th percentile (circle) and log-normal mean (triangle) tumor size for each sgRNA species within the pool. These two metrics are reported in terms of tumor size relative to tumors initiated with inert sgRNAs (driven by oncogenic KRAS alone). Data are shown for both Cas9-deficient (*KT*; top) or proficient (*KT;H11*$^{LSL\text{-}Cas9}$) settings. Fill color reflects whether tumor size is significantly different from that of tumors initiated with inert sgRNAs, gray fill indicates no significant difference. Error bars indicate 95% confidence intervals centered on the mean relative tumor size.

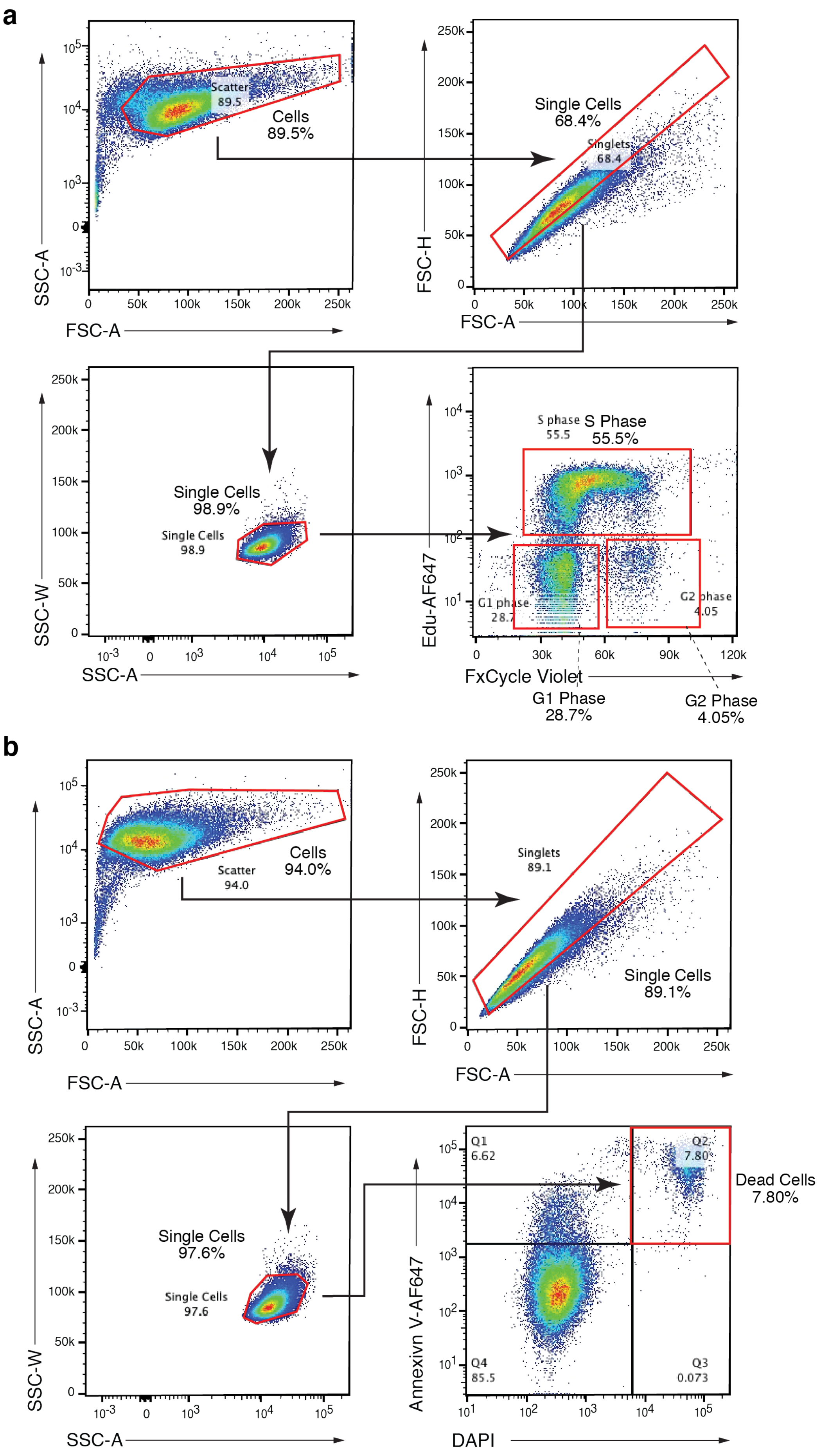

Supplementary Figure 19. Flow cytometry gating strategy for cell cycle and cell death analyses.

a. Gating strategy to determine the fraction of cells within S, G1, and G2 phases of cell cycle using EdU incorporation and FxCycle Violet staining.
b. Gating strategy to determine the fraction of dead cells using Annexin V and DAPI staining..

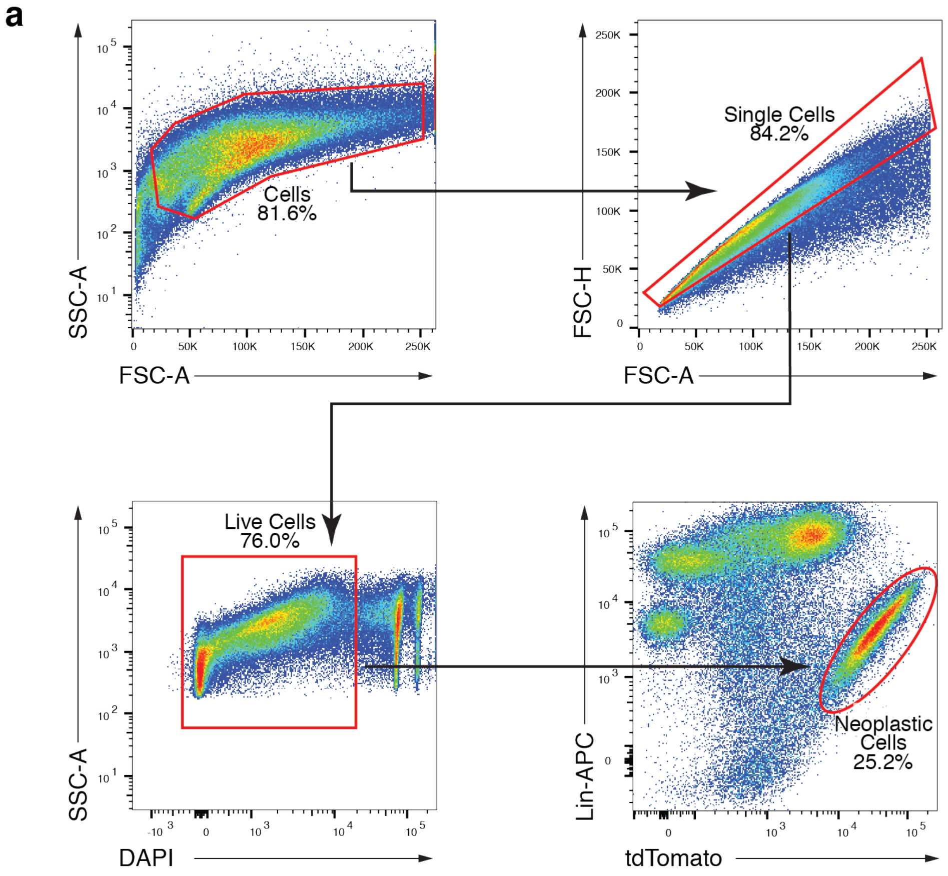

Supplementary Figure 20. FACS gating strategy for isolating tdTomato^positive^ neoplastic cell isolation from mouse lung tumors.

a. Sequential gating on dissociated lung tumor cells to enable FACS-based isolation of tdTomato^positive^ neoplastic cells. Lin = lineage markers: CD45, CD31, F4/80, Ter119 (for the exclusion of hematopoietic and endothelial cells).

Supplementary Table 1. Summary of sgRNAs used for the interrogation of tumor-suppressive function *in vivo*.

Experiment	Gene Target	Version	sgRNA Sequence	Class	Source
Primary/Secondary Screen	Neo	1	TCATGGCTGATGCAATGCGG	Inert	Chiou, Winters, *et al.* 2015
Primary/Secondary Screen	Neo	2	GATATTGCTGAAGAGCTTGG	Inert	Rogers, McFarland, Winters, *et al.* 2017
Primary/Secondary Screen	Neo	3	GAATAGCCTCTCCACCCAAG	Inert	Rogers, McFarland, Winters, *et al.* 2017
Primary/Secondary Screen	Non-Targeting	1	GCGAGGTATTCGGCTCCGCG	Inert	Chiou, Winters, *et al.* 2015
Primary/Secondary Screen	Non-Targeting	2	GACGTAGCCTTCCGAAATAT	Inert	Rogers, McFarland, Winters, *et al.* 2017
Primary/Secondary Screen	*Lkb1*	1	GTGGTGGGCCGCAGTCACAA	Positive Control	Chiou, Winters, *et al.* 2015
Primary/Secondary Screen	*Lkb1*	2	GCCACTCTCTGACCTACTCCG	Positive Control	Cai, Chew, Li, *et al.* 2021
Primary Screen	*Sik1*	1	GCTGGACACAGCTATCAGCG	Positive Control	Murray, *et al.* 2019
Primary Screen	*Sik3*	1	GTTCAAACAGATCGTCACAG	Positive Control	Murray, *et al.* 2019
Primary/Secondary Screen	*Cebpa*	1	GTGCTCGCAGATGCCGCCCAG	Experimental	This Paper
Secondary Screen	*Cebpa*	2	GCATCAGCGCCTACATCGACC	Experimental	This Paper
Primary/Secondary Screen	*Cebpb*	1	GATGGGTCTAAAGGCGGCGGG	Experimental	This Paper
Secondary Screen	*Cebpb*	2	GTGCTCGCCAATGGCCGGCT	Experimental	This Paper
Primary/Secondary Screen	*Cebpd*	1	GCTCAAGCGCGAACCCGACTG	Experimental	This Paper
Secondary Screen	*Cebpd*	2	GTCGTACATGGCAGGAGTCG	Experimental	This Paper
Primary Screen	*Aass*	1	GACTTACGTCTCGCACCACTG	Experimental	This Paper
Primary Screen	*Acoxl*	1	GACTGTCGTGAATGCACCGG	Experimental	This Paper
Primary Screen	*Acss1*	1	GAAGGGCGCTCTGGACAATCG	Experimental	This Paper
Primary Screen	*Aldh4a1*	1	GCAACTGGTACTGTATATCGG	Experimental	This Paper
Primary Screen	*Aox3*	1	GCAGATAATGGAAACCCTGGG	Experimental	This Paper
Primary Screen	*Car8*	1	GCAATGATGGCGATACCATG	Experimental	This Paper
Primary Screen	*Ces1d*	1	GTTAACAGTGTTCACCACAGG	Experimental	This Paper
Primary Screen	*Cldn8*	1	GAAGTGCACCAGATGCACGG	Experimental	This Paper
Primary Screen	*Cyb5r1*	1	GCTGCGATTGCTAGACAAGA	Experimental	This Paper
Primary Screen	*Echdc3*	1	GAGGCCGTGATTATCACGCTG	Experimental	This Paper
Primary Screen	*Gabrp*	1	GTGCCGCTCACCTCTGAGTG	Experimental	This Paper
Primary Screen	*Gas6*	1	GCTATCACCTGAATCTCACCG	Experimental	This Paper
Primary Screen	*Glul*	1	GCCTTCAGGCCCGTATTACTG	Experimental	This Paper
Primary Screen	*Gpnmb*	1	GAGAGAGCACAACCAATTACG	Experimental	This Paper
Primary Screen	*Hadh*	1	GCAAGCTTCATCATAGGCACG	Experimental	This Paper
Primary Screen	*Hnmt*	1	GAGCTTCCGGGCATAATAGCA	Experimental	This Paper
Primary Screen	*Lyz2*	1	GCAAGAGCTGTGAATGCCTGT	Experimental	This Paper
Primary Screen	*Mat1a*	1	GAGAGATAAATACGCACCTGG	Experimental	This Paper
Primary Screen	*Mccc1*	1	GCTTCGGCAGACATGTCTACG	Experimental	This Paper
Primary Screen	*Pccb*	1	GCTTGTGCTGCGCGTCGATG	Experimental	This Paper
Primary Screen	*Sh3bgrl*	1	GCAATGAATGCCAGTACCGTG	Experimental	This Paper
Primary Screen	*Snd1*	1	GAGTATGGGATGATCTACCT	Experimental	This Paper
Primary Screen	*St6galnac2*	1	GCCGCCGAGGAGTACAGCATG	Experimental	This Paper
Primary Screen	*Tst*	1	GAAGACGCGTCCAGCACCCGA	Experimental	This Paper
Primary Screen	*Vnn1*	1	GATAGGAAGTCCAGTTCACCG	Experimental	This Paper

Supplementary Table 2. Primer sequences for cloning individual sgRNAs and assembling multi-sgRNA Lenti-Cre vectors.

Primer	Sequence
pLL3 Site-Directed Mutagenesis Forward	(G)+protospacer+GTTTAAGAGCTATGCTGGAAACAG
pLL3 Site-Directed Mutagenesis Reverse	GGTGTTTCGTCCTTTCCAC
pMJ117 Site-Directed Mutagenesis Forward	(G)+protospacer+GTTTCAGAGCTAAGCACAAGAGT
pMJ117 Site-Directed Mutagenesis Universal Reverse	CATGTTTCTGGCTTTCCACAAG
pMJ179 Site-Directed Mutagenesis Forward	(G)+protospacer+GTTTGAGAGCTAAGCAGAAAGCT
pMJ179 Site-Directed Mutagenesis Universal Reverse	CAACAAGGTGGTTCTCCAAGG
pMJ114 Site-Directed Mutagenesis Forward	(G)+protospacer+GTTTAAGAGCTAAGCTGGAAACAG
pMJ114 Site-Directed Mutagenesis Universal Reverse	CATAAACATGGCCTCTCAAGAACT
pLL3 Linearization for Gibson asssembly Forward	GTTTGGCGCGCCTATGGCTTAA
pLL3 Linearization for Gibson asssembly Reverse	ACAGTTTAAACAGTTCCTGCAGGGC
hU6-sgRNA Gibson Amplification Forward	**ATAGGCGCGCCAAAC**AAAAAAAGCACCCGACTCGG
hU6-sgRNA Gibson Amplification Reverse	**GCTCGAATCTACACTCAGC**TATG
mU6-sgRNA Gibson Amplification Forward (Dual)	**AACTGTTTAAACTGT**GATCCGACGCGCCATCTCTA
mU6-sgRNA Gibson Amplification Forward (Triple)	**CCAGTCTGACTATCACGATT**GA
mU6-sgRNA Gibson Amplification Reverse	**GCTGAGTGTAGATTCGAGC**AAA
bU6-sgRNA Gibson Amplification Forward	**AACTGTTTAAACTGT**CGTGACCGAGCTTGTCTG
bU6-sgRNA Gibson Amplification Reverse	**AATCGTGATAGTCAGACTGG**AAAAA

*** Bold sequences denote regions of homology for Gibson assembly**
