# Supplementary material for: LKB1 drives stasis and C/EBP-mediated reprogramming to an alveolar type II fate in lung cancer
Source: Nat Commun. 2022 Feb 28;13:1090. doi: 10.1038/s41467-022-28619-8 (PMC8885825; doi:10.1038/s41467-022-28619-8)
Supplement: Supplementary file 3 — Description of Additional Supplementary Files [file 41467_2022_28619_MOESM3_ESM.pdf]

Description of Additional Supplementary Files

File Name: Supplementary Data 1
Description: RLE-normalized (DESeq2) counts for *Lkb1*^{XTR} RNA-seq experiment, including *Trp53* wild-type and deficient tumors.

File Name: Supplementary Data 2
Description: Differential expression analysis results for the comparison of *Lkb1*-restored tumors to *KT* tumors. *P* values were obtained using the Wald test and corrected for multiple hypothesis testing using the Benjamini-Hochberg procedure.

File Name: Supplementary Data 3
Description: Differential expression analysis results for the comparison of *Lkb1*-restored tumors to non-restored tumors. *P* values were obtained using the Wald test and corrected for multiple hypothesis testing using the Benjamini-Hochberg procedure.

File Name: Supplementary Data 4
Description: Differential expression analysis results for the comparison of non-restored tumors to *KT* tumors. *P* values were obtained using the Wald test and corrected for multiple hypothesis testing using the Benjamini-Hochberg procedure.

File Name: Supplementary Data 5
Description: Differential expression analysis results for the comparison of *Lkb1*-restored tumors to non-restored tumors in the absence of *Trp53*. *P* values were obtained using the Wald test and corrected for multiple hypothesis testing using the Benjamini-Hochberg procedure.

File Name: Supplementary Data 6
Description: Total protein intensities for *Lkb1*^{XTR} shotgun mass spectrometry experiment.

File Name: Supplementary Data 7
Description: Differential abundance analysis results for the comparison of *Lkb1*-restored tumors to non-restored tumors by shotgun mass spectrometry. *P* values were obtained using the moderated t-test and corrected for multiple hypothesis testing using the Benjamini-Hochberg

File Name: Supplementary Data 8
Description: RLE-normalized (DESeq2) counts for C/ebps and *Lkb1* knockout RNA-seq experiment.

File Name: Supplementary Data 9
Description: Differential expression analysis results for the comparison of sgCebps tumors to sgInert tumors. *P* values were obtained using the Wald test and corrected for multiple hypothesis testing using the Benjamini-Hochberg procedure.

File Name: Supplementary Data 10
Description: Differential expression analysis results for the comparison of sg*Lkb1* tumors to sgInert tumors. *P* values were obtained using the Wald test and corrected for multiple hypothesis testing using the Benjamini-Hochberg procedure.

File Name: Supplementary Data 11
Description: Differential expression analysis results for the comparison of sgCebps tumors to sg*Lkb1* tumors. *P* values were obtained using the Wald test and corrected for multiple hypothesis testing using the Benjamini-Hochberg procedure.
