# Supplementary material for: LKB1 drives stasis and C/EBP-mediated reprogramming to an alveolar type II fate in lung cancer
Source: Nat Commun. 2022 Feb 28;13:1090. doi: 10.1038/s41467-022-28619-8 (PMC8885825; doi:10.1038/s41467-022-28619-8)
Supplement: Supplementary file 15 — Reporting Summary [file 41467_2022_28619_MOESM15_ESM.pdf]

nature portfolio

Corresponding author(s): Monte M. Winslow

Last updated by author(s): Dec 28, 2021

# Reporting Summary

Nature Portfolio wishes to improve the reproducibility of the work that we publish. This form provides structure for consistency and transparency in reporting. For further information on Nature Portfolio policies, see our Editorial Policies and the Editorial Policy Checklist.

## Statistics

For all statistical analyses, confirm that the following items are present in the figure legend, table legend, main text, or Methods section.

| n/a | Confirmed | |
|---|---|---|
| ☐ | ☒ | The exact sample size (*n*) for each experimental group/condition, given as a discrete number and unit of measurement |
| ☐ | ☒ | A statement on whether measurements were taken from distinct samples or whether the same sample was measured repeatedly |
| ☐ | ☒ | The statistical test(s) used AND whether they are one- or two-sided<br>*Only common tests should be described solely by name; describe more complex techniques in the Methods section.* |
| ☒ | ☐ | A description of all covariates tested |
| ☐ | ☒ | A description of any assumptions or corrections, such as tests of normality and adjustment for multiple comparisons |
| ☐ | ☒ | A full description of the statistical parameters including central tendency (e.g. means) or other basic estimates (e.g. regression coefficient) AND variation (e.g. standard deviation) or associated estimates of uncertainty (e.g. confidence intervals) |
| ☐ | ☒ | For null hypothesis testing, the test statistic (e.g. *F*, *t*, *r*) with confidence intervals, effect sizes, degrees of freedom and *P* value noted<br>*Give P values as exact values whenever suitable.* |
| ☒ | ☐ | For Bayesian analysis, information on the choice of priors and Markov chain Monte Carlo settings |
| ☒ | ☐ | For hierarchical and complex designs, identification of the appropriate level for tests and full reporting of outcomes |
| ☐ | ☒ | Estimates of effect sizes (e.g. Cohen's *d*, Pearson's *r*), indicating how they were calculated |

*Our web collection on statistics for biologists contains articles on many of the points above.*

## Software and code

Policy information about availability of computer code

| | |
|---|---|
| Data collection | qRT-PCR data was acquired Sequence Detection Systems Software v2.4.1 in Absolute Quantitation mode.<br>Serial measurements of tumor size were captured by µCT using the Trifoil CT eXplore CT120 with respiratory gating and volumes were reconstructed using MicroView v.2.5.0 (Parallax Innovations).<br><br>The frequency of H3P- and Ki-67-positive nuclei from immunohistochemical staining was quantified in ImageJ v2.1.0. Measures of tumor area and tumor size were also captured in ImageJ v2.1.0. |
| Data analysis | For Tuba-seq analysis, Python 3.6 and R 3.6 were used to analyze the data, and code is available at https://github.com/lichuan199010/Tuba-seq-analysis-and-summary-statistics.<br>For bulk RNA-seq, reads were aligned to the mm10 mouse genome using STAR (v2.6.1d). Estimates of transcript abundance were obtained using RSEM (v1.2.30). The differentially expression analysis was performed with DESeq2 (v1.26.0). Gene set enrichment was performed with GSEA (v3.0) (Subramanian, et al. 2005). Enriched GO terms were clustered in Cytoscape (v3.8.2) by importing GSEA using the EnrichmentMap plugin. Networks were ported to R using ggraph (v2.0.4) and clusters of related GO terms were defined using the edge betweenness community detection algorithm in igraph (v1.2.6). K-means clusters were defined in ComplexHeatmap (v2.2.0) and GO term enrichment analysis was performed using compareCluster in ClusterProfiler (v3.14.3). Motif enrichment was performed using Pscan (Zambelli, et al. 2009, PMID: 19487240) [http://159.149.160.88/pscan/].<br><br>For single cell RNA-seq, reads were aligned to the mm10 genome and feature counts were obtained using Cell Ranger (v3.0.2). Feature-barcode matrices were then imported into R using Seurat (v3.2.0). The Louvain algorithm for community detection was employed using the |

Seurat (v3.2.0) implementation. For cell-type prediction within the total cell (non-sorted) dataset, SingleR (v1.4.1) was used with the Tabula Muris lung dataset as a reference. For the total cell dataset, clusters (defined by Louvain algorithm) were aggregated into pseudobulk samples and differential expression analysis between restored and non-restored samples was performed using muscat (v1.4.0). Trajectory inference analysis was performed using Monocle3 (v0.2.1). For RNA velocity analysis, spliced, unspliced, and ambiguous matrices were generated using velocyto (v0.17.17). RNA velocity analysis was then performed using scvelo (v.0.2.3).

Shotgun mass spectrometry data were analyzed processing Bruker raw data files with msfragger using the tool FragPipe. Peptide and protein identifications were validated using PeptideProphet. Quantitation was done using IonQuant. Razor intensities were analyzed and differential expression analyses were performed using DEP (v1.8.0) and limma (v3.42.2).

For manuscripts utilizing custom algorithms or software that are central to the research but not yet described in published literature, software must be made available to editors and reviewers. We strongly encourage code deposition in a community repository (e.g. GitHub). See the Nature Portfolio guidelines for submitting code & software for further information.

## Data

Policy information about availability of data

All manuscripts must include a data availability statement. This statement should provide the following information, where applicable:

- Accession codes, unique identifiers, or web links for publicly available datasets
- A description of any restrictions on data availability
- For clinical datasets or third party data, please ensure that the statement adheres to our policy

Next-generation sequencing data for the Tuba-Seq and RNA-Seq (bulk and single-cell) experiments are accessioned under the GSE179560 SuperSeries at NCBI Gene Expression Omnibus [https://www.ncbi.nlm.nih.gov/geo/query/acc.cgi?acc=GSE179560]. Shotgun proteomics data are accessioned under PXD026738 at PRIDE [https://www.ebi.ac.uk/pride/archive/projects/PXD026738]. Lenti-sgRNA/Cre plasmids generated in this study are available through the Winslow Lab plasmid collection on Addgene [https://www.addgene.org/Monte_Winslow/]. Lkb1XTR/XTR mice generated in this paper are available at The Jackson Laboratory (Stock no. 034052). Lkb1XTR/XTR mouse lung cancer cell lines are available from the corresponding author upon request. JASPAR 2018 non-redundant [https://jaspar.genereg.net/api/v1/live-api/] and TRANSFAC [http://cisbp.ccbr.utoronto.ca/index.php] databases are publicly available and accessible via Pscan [http://159.149.160.88/pscan/]. Previously published gene expression data derived from lung tumors in genetically engineered mice are available under accession numbers GSE6135 [https://www.ncbi.nlm.nih.gov/geo/query/acc.cgi?acc=GSE6135], GSE21581 [https://www.ncbi.nlm.nih.gov/geo/query/acc.cgi?acc=GSE21581], GSE69552 [https://www.ncbi.nlm.nih.gov/geo/query/acc.cgi?acc=GSE69552], and GSE133714 [https://www.ncbi.nlm.nih.gov/geo/query/acc.cgi?acc=GSE133714] at NCBI Gene Expression Omnibus. Lung cell identity gene expression signatures were derived from publicly available single-cell RNA-seq datasets, including Tabula Muris & Tabula Muris Senis [https://www.synapse.org/#!Synapse:syn21560554]; Mouse Cell Atlas [http://bis.zju.edu.cn/MCA/dpline.html?tissue=Lung]; Strunz, et al. 2020 [https://github.com/theislab/2019_Strunz]; Little, et al. 2019 -- Gene Expression Omnibus: GSE129584 [https://www.ncbi.nlm.nih.gov/geo/query/acc.cgi?acc=GSE129584]; Angelidis, et al. 2019 [https://github.com/gtsitsiridis/lung_aging_atlas]; Guo et al., 2019 -- Gene Expression Omnibus: GSE122332 [https://www.ncbi.nlm.nih.gov/geo/query/acc.cgi?acc=GSE122332]; Treutlein, et al. 2014 [https://hemberg-lab.github.io/scRNA.seq.datasets/mouse/tissues/]. Source data are provided with this paper.

# Field-specific reporting

Please select the one below that is the best fit for your research. If you are not sure, read the appropriate sections before making your selection.

☒ Life sciences ☐ Behavioural & social sciences ☐ Ecological, evolutionary & environmental sciences

For a reference copy of the document with all sections, see nature.com/documents/nr-reporting-summary-flat.pdf

# Life sciences study design

All studies must disclose on these points even when the disclosure is negative.

| | |
|---|---|
| Sample size | No statistical methods were used to predetermine mouse cohort size. Cohort sizes were determined to account for statistical noise, based on previous experience with the Tuba-seq method (Rogers, et al.; PMID: 28530655). The cohort size for other experiments was determined based on our previous publications (Murray, et al.; PMID: 31350327; Robles-Oteiza, et al.; PMID: 26537451). |
| Data exclusions | No data were excluded from the manuscript. |
| Replication | Mouse studies were not replicated, but they included sufficient sample sizes to account for biological variability. Replication in mouse experiments is not standard given their time- and cost-intensive nature. |
| Randomization | For all experiments, mice were randomly grouped to avoid bias. |
| Blinding | Investigators were not blinded for the analysis of experiments as the genotypes of experimental and control mice were known to the investigators. The qRT-PCR, Western blotting, histology, and immunohistochemistry experiments were reviewed by at least two independent persons. |

# Reporting for specific materials, systems and methods

We require information from authors about some types of materials, experimental systems and methods used in many studies. Here, indicate whether each material, system or method listed is relevant to your study. If you are not sure if a list item applies to your research, read the appropriate section before selecting a response.

## Materials & experimental systems

| n/a | Involved in the study | |
|---|---|---|
| ☐ | ☒ | Antibodies |
| ☐ | ☒ | Eukaryotic cell lines |
| ☒ | ☐ | Palaeontology and archaeology |
| ☐ | ☒ | Animals and other organisms |
| ☒ | ☐ | Human research participants |
| ☒ | ☐ | Clinical data |
| ☒ | ☐ | Dual use research of concern |

## Methods

| n/a | Involved in the study | |
|---|---|---|
| ☒ | ☐ | ChIP-seq |
| ☐ | ☒ | Flow cytometry |
| ☒ | ☐ | MRI-based neuroimaging |

# Antibodies

Antibodies used

The following antibodies were used for western blot on mouse protein lysates:
LKB1 (Cell Signaling Technology, Catalog Number: 13031, Clone: D60C5F10); GAPDH (Cell Signaling Technology, Catalog Number: 5174, Clone: D16H11); HRP-conjugated anti-rabbit (Santa Cruz Biotechnology: sc-2004); HRP-conjugated anti-mouse (Santa Cruz Biotechnology: sc-2005).

The following antibodies were used for immunohistochemistry on formalin-fixed, paraffin-embedded mouse tissues:
Cleaved Caspase 3 (Cell Signaling Technologies: 9661S, polyclonal); phosphorylated Histone H3 Serine 10 (Cell Signaling Technologies: 9701S, polyclonal); Ki-67 (BD Biosciences: 550609, Clone: B56); NKX2-1 (Abcam: ab76013, Clone: EP1584Y); HMGA2 (Biocheck: 59170AP, polyclonal).

The following antibodies were used for FACS on dissociated cells from mouse lung tumors:
APC-conjugated anti-CD45 (BioLegend: 103112, Clone: 30-F11), APC-conjugated anti-CD31 (BioLegend: 102402, Clone: 390), APC-conjugated anti-F4/80 (BioLegend: 123116, Clone: BM8), and APC-conjugated anti-Ter119 (BioLegend: 116212, Clone: TER-119).

Validation

LKB1 (Cell Signaling Technology, Catalog Number: 13031, Clone: D60C5F10) -- Previously validated with in vivo CRISPR/Cas9-mediated knockout of Lkb1 (Chiou et al., 2015, PMID: 26178787).
GAPDH (Cell Signaling Technology, Catalog Number: 5174, Clone: D16H11) -- validated by manufacturer and cited in publications, suitable for western blot.
Cleaved Caspase 3 (Cell Signaling Technologies: 9661S, polyclonal) -- validated by manufacturer; caspase 3 cleavage induced in cell lines via treatment with staurosporine or cytochrome c, suitable for IHC.
phosphorylated Histone H3 Serine 10 (Cell Signaling Technologies: 9701S, polyclonal) -- validated by manufacturer, histone H3 phosphorylation induced in cell lines via treatment with serum, suitable for IHC.
Ki-67 (BD Biosciences: 550609, Clone: B56) -- validated by manufacturer, labeling of Ki-67+ cells is blocked by pre-treatment with another clone against Ki-67 (MIB1), suitable for IHC.
NKX2-1 (Abcam: ab76013, Clone: EP1584Y) -- Previously validated by IHC in lung tumors of KP and KP;Nkx2-1flox/flox mice (Mollaoglu et al., 2018, PMID: 30332632)
HMGA2 (Biocheck: 59170AP, polyclonal) -- Previously validated by IHC in pancreatic ductal adenocarcinomas within KP and KP;Nkx2-1flox/flox mice (Chiou et al., 2018, PMID: 30228296)
APC-conjugated anti-CD45 (BioLegend: 103112, Clone: 30-F11) -- validated by manufacturer and cited in publications, stained C57BL6/J mouse splenocytes.
APC-conjugated anti-CD31 (BioLegend: 102402, Clone: 390) -- validated by manufacturer and cited in publications, stained C57BL6/J mouse splenocytes.
APC-conjugated anti-F4/80 (BioLegend: 123116, Clone: BM8) -- validated by manufacturer and cited in publications, stained peritoneal macrophages in BALB/c mice injected with thioglycolate.
APC-conjugated anti-Ter119 (BioLegend: 116212, Clone: TER-119) -- validated by manufacturer and cited in publications, stained erythroid population present among C57BL6/J mouse bone marrow cells.

# Eukaryotic cell lines

Policy information about cell lines

Cell line source(s)

293T cells were acquired from ATCC (CRL-3216). LSL-YFP MEFs were obtained from Dr. Alejandro Sweet-Cordero (UCSF). Mouse lung cancer cell lines 394T4 (KP), 3406 (KP;Lkb1XTR/XTR), 2841T6 (KP;Lkb1XTR/XTR;FLPo-ERT2), 3841T4 (KP;Lkb1XTR/XTR;FLPo-ERT2), and 2804T5B (KP;Lkb1XTR/XTR;FLPo-ERT2) were derived from primary lung tumors as described in the Methods section.

Authentication

293T cells used for lentivirus production were validated by verifying that high-titer lentivirus production was possible. LSL-YFP cells were validated by measuring Cre-dependent activation of YFP expression.

Mycoplasma contamination

All cell lines included in this study tested negative for mycoplasma contamination (Lonza MycoAlert Mycoplasma Detection Kit, #LT07-218).

Commonly misidentified lines
(See ICLAC register)

We have not used any commonly misidentified cell lines in this study.

## Animals and other organisms

Policy information about studies involving animals; ARRIVE guidelines recommended for reporting animal research

| | |
|---|---|
| Laboratory animals | Tumor studies involving genetically engineered mice included males and females (Mus musculus) on a C57BL/6J:129 mixed background aged 6 to 12 weeks. KrasLSL-G12D (The Jackson Laboratory: stock no. 008179), p53flox (The Jackson Laboratory: stock no. 08462), H11LSL-Cas9 (The Jackson Laboratory: stock no. 027632), Rosa26FLPo-ERT2 (The Jackson Laboratory: stock no. 018906), and Rosa26LSL-tdTomato (ai9 and ai14 alleles; The Jackson Laboratory: stock no. 007909 & 007908), Lkb1XTR (The Jackson Laboratory: stock no. 034052). NOD/SCID/gammaC mice (The Jackson Laboratory: stock no. 005557) used as allograft recipients included males and females aged 6-10 weeks. Mice were housed at 22°C ambient temperature with 40% humidity and a 12-hour light/dark cycle. |
| Wild animals | This study did not involve wild animals. |
| Field-collected samples | This study did not involve field-collected samples. |
| Ethics oversight | Mice were maintained within Stanford University's SIM1 Barrier Facility according to practices prescribed by the NIH and the Institutional Animal Care and Use Committee (IACUC) at Stanford University. Additional accreditation of Stanford University Research Animal Facility was provided by the Association for Assessment and Accreditation of Laboratory Animal Care (AAALAC). Protocols employed in this study were approved by the Administrative Panel on Laboratory Animal Care (APLAC) at Stanford University (Protocol #26696). |

Note that full information on the approval of the study protocol must also be provided in the manuscript.

## Flow Cytometry

### Plots

Confirm that:

☒ The axis labels state the marker and fluorochrome used (e.g. CD4-FITC).

☒ The axis scales are clearly visible. Include numbers along axes only for bottom left plot of group (a 'group' is an analysis of identical markers).

☒ All plots are contour plots with outliers or pseudocolor plots.

☒ A numerical value for number of cells or percentage (with statistics) is provided.

### Methodology

| | |
|---|---|
| Sample preparation | For cell cycle analysis, EdU-treated cells were washed, trypsinized, and subsequently stained with AF647 (Thermo Fisher Scientific C10424) and FxCycle Violet (Thermo Fisher Scientific F10347) according to manufacturer's guidelines for double staining with the Click-iT system and FxCycle dyes. For cell death analysis, detached dead cells were collected within the culture supernatant prior to washing and trypsinization of remaining attached cells. Supernatant dead cells were aggregated with detached viable cells prior to staining with AF647-conjugated Annexin V (Thermo Fisher, A23204) and DAPI.<br><br>For sorting of lung tumor cells and specifically neoplastic cells, micro-dissected lung tumors were dissociated with collagenase IV, dispase, and trypsin at 37C for 30 minutes. The digestion buffer was then neutralized with cold L-15 media (Thermo Fisher Scientific: 21083027) containing 5% FBS (Gemini Bio) and DNaseI (Sigma-Aldrich: DN25. Dissociated cells were treated with ACK Lysis Buffer (Thermo Fisher Scientific: A1049201) and resuspended in PBS containing 2 mM EDTA (Promega: V4233), 2% FBS, and DNase I. For the isolation of neoplastic cells, dissociated cells were stained with DAPI and antibodies against CD45 (BioLegend: 103112), CD31 (BioLegend: 303116), F4/80 (BioLegend: 123116), and Ter119 (BioLegend: 116212) to exclude hematopoietic and endothelial cells. |
| Instrument | Flow cytometry analysis was performed on LSRII Analyzers (BD Biosciences). Sorting of total viable lung tumor cells and neoplastic cells was performed on FACSAria™ sorters (BD Biosciences). |
| Software | Data were acquired using either BD FACSDiva software (BD Biosciences). Data were analyzed using FlowJo v10. |
| Cell population abundance | Neoplastic cells are sorted using 4-way purity mode to maximize purity. Purity of sorted neoplastic cells was not directly assessed. Contamination of sorted neoplastic cell samples with hematopoeitic cells, endothelial cells, and fibroblasts was minimal as determined by single cell RNA-seq. |

Gating strategy

For flow cytometry analysis, cells were gated by FSC area vs. SSC area, and singlets were gated by FSC area vs FSC height, followed by SSC area vs. SSC height. For cell cycle analyses, all EdU-AF647+ cells were considered cells in S phase. G1 cells were EdU-AF647- with 2N DNA content, and G2/M cells were EdU-AF647- with 4N DNA content. For cell death analysis, cells that were Annexin V-AF647+ and DAPI+ were identified as dead cells.

For sorting of lung tumor cells and specifically neoplastic cells, cells were gated by FSC area vs. SSC area, and singlets were gated by FSC area vs FSC height. Viable cells were gated by DAPI vs. SSC area and neoplastic cells were subsequently gated by tdTomato vs. APC-labeled lineage markers.

☒ Tick this box to confirm that a figure exemplifying the gating strategy is provided in the Supplementary Information.
